# The RESP AI model accelerates the identification of tight-binding antibodies

**DOI:** 10.1038/s41467-023-36028-8

**Published:** 2023-01-28

**Authors:** Jonathan Parkinson, Ryan Hard, Wei Wang

**Affiliations:** 1 Department of Chemistry and Biochemistry, University of California, San Diego, La Jolla, CA 92093-0359 USA; 2 Department of Cellular and Molecular Medicine, University of California, San Diego, La Jolla, CA 92093-0359 USA

**Keywords:** Protein design, Protein function predictions

## Abstract

High-affinity antibodies are often identified through directed evolution, which may require many iterations of mutagenesis and selection to find an optimal candidate. Deep learning techniques hold the potential to accelerate this process but the existing methods cannot provide the confidence interval or uncertainty needed to assess the reliability of the predictions. Here we present a pipeline called RESP for efficient identification of high affinity antibodies. We develop a learned representation trained on over 3 million human B-cell receptor sequences to encode antibody sequences. We then develop a variational Bayesian neural network to perform ordinal regression on a set of the directed evolution sequences binned by off-rate and quantify their likelihood to be tight binders against an antigen. Importantly, this model can assess sequences not present in the directed evolution library and thus greatly expand the search space to uncover the best sequences for experimental evaluation. We demonstrate the power of this pipeline by achieving a 17-fold improvement in the *K*_D_ of the PD-L1 antibody Atezolizumab and this success illustrates the potential of RESP in facilitating general antibody development.

## Abstract (web summary)

High-affinity antibodies are often identified through directed evolution but deep leaning methods hold great promise. Here the authors report RESP, a pipeline for efficient identification of high affinity antibodies, and apply this to the PD-L1 antibody Atezolizumab.

## Introduction

Monoclonal antibodies are among the most successful of biological therapeutics^1^. Despite their impressive versatility and specificity, development of therapeutic antibodies continues to pose a variety of complex challenges. Typically initial hits have insufficient affinity and their binding must first be improved through in vitro affinity maturation, whereby repeated rounds of mutagenesis and selection for antibodies with improved affinity are performed^2,3^. This process is frequently time-intensive and may take months to complete^3^, and cannot simultaneously optimize for other desirable properties like good solubility and low immunogenicity^4,5^. Computational techniques that could aid in the faster identification of high-affinity antibodies with desirable properties would likely accelerate this process.

Traditional computational approaches to antibody binding affinity rely on estimation of free energy^3,6,7^. These are often limited by high computational cost, low throughput and the limited reliability of the free energy estimates generated by these methods^7,8^. Alternatively, machine learning techniques have been applied to both protein engineering and a variety of tasks in antibody design^9–25^.

Machine learning-based approaches face at least two major challenges. The first one is the lack of estimated uncertainty in the predictions of the binding affinities or other properties. As the training data can only ever cover a small fraction of the sequence space, machine learning models typically perform poorly when asked to extrapolate far beyond the bounds of their training set^26^. Deep learning models, while flexible and powerful, typically (outside of specific architectures) do not provide confidence intervals or estimates of uncertainty in their predictions^27^. Gaussian process models have been suggested as an alternative that does provide well-calibrated confidence intervals^28^, but they scale poorly to large datasets without the use of approximations and are often infeasible for datasets larger than 5000 sequences^29^.

The second key challenge is the selection of an appropriate representation for the input. Many different encoding schemes for proteins have been described in the literature, including most recently ones adopting language models; some of these are antibody-specific and some are general to protein sequences^30–35^. There is however little consensus on which of these is most appropriate for a given problem. The classic one-hot encoding scheme is simple to implement but unnecessarily high-dimensional and uninformative, since every amino acid is treated as being completely different from each other^9,36,37^.

In this paper, we develop an easy to implement machine learning-assisted pipeline for the identification of high-affinity antibodies that addresses these challenges. We train an autoencoder model on over 3 million B-cell receptor sequences and show that this learned representation provides better results for a task of interest than state-of-the-art embedding schemes. Next, we develop a Bayesian neural network trained to perform ordinal regression to model the relationship between sequence and binding affinity or off-rate using the directed evolution data. Importantly, this model provides an estimate of the uncertainty in its predictions that cannot be achieved by the current deep learning methods. To benchmark this approach, we show it achieves competitive accuracy on a literature dataset^11^, while providing useful uncertainty information not provided by the deep learning model in the original associated study. Finally, we perform in silico mutagenesis using a simulated annealing strategy to explore sequences not present in the mutation libraries and assess their binding affinities for experimental evaluation. Together, the autoencoder, the Bayesian network for ordinal regression and the search strategy form the key computational components of our RESP pipeline.

As a proof of concept to demonstrate the power of RESP, we attempted to improve the affinity of a well-known antibody to a well-studied antigen by engineering mutants of the heavy chain of Atezolizumab (brand name Tecentriq) with improved affinity for programmed death ligand 1 (PD-L1)^38,39^. We randomized a large portion of the Atezolizumab heavy chain sequence, binned the mutants using yeast display and FACS, and sampled each bin for sequencing. The mutant Atezolizumab sequence data was converted to a low-dimensional representation using the autoencoder model, and the encoded mutant Atezolizumab sequences were used to train a Bayesian ordinal regression model that scores each sequence on the probability it will be a strong binder to PD-L1. Finally, a modified simulated annealing algorithm was used to select sequences for testing. We show here that our pipeline discovered a panel of Atezolizumab scFv mutants with improved off-rates towards PD-L1, and one characterized mutant displayed about a 10-fold decrease in off-rate and 17-fold improvement in *K*_D_ between human PD-L1 and Atezolizumab. Our method should be useful as a general approach for improving antibody-antigen interactions while reducing the experimental effort to do so. The mutant we discovered could be a useful reagent for treating PD-L1 positive tumors.

## Results

### Overview of the RESP pipeline

The RESP pipeline comprises four key components. First, we have developed a simple encoding scheme in which an autoencoder is designed to learn representations that incorporate features distinguishing human B-cell receptor (BCR) sequences from closely related sequences. We show this learned representation enables more efficient and accurate modeling of trends in fluorescence-activated cell sorting (FACS) data than provided by other popular learned representations of protein and antibody sequences (see Table 1). This part of the pipeline is general to any antibody sequence and this representation can be reused for any project.

**Table 1:** Performance comparisons across different encoding types and model architectures for classification performance on the Atezolizumab dataset

Encoding type	Model type	Num hidden layer weights	MCC on 5× CV for all class classification	AUC-ROC on 5× CV for RH03 vs rest classification	MCC on the test set for all-class classification
One-hot (default)	Bayesian NN w/ ordinal regression (BNN-OR)	84,090	0.717 ± 0.009	0.967 ± 0.001	0.721
Autoencoder	BNN-OR	12,810	0.69 ± 0.006	0.966 ± 0.002	0.703
UniRep	BNN-OR	114,930	0.62 ± 0.01	0.947 ± 0.003	0.638
ProtVec	BNN-OR	3930	0.641 ± 0.003	0.852 ± 0.003	0.650
ESM-1b	BNN-OR	39,330	0 (model did not converge)	–	–
AbLang	BNN-OR	23,970	0.636 ± 0.01	0.96 ± 0.002	0.664
AntiBertY	BNN-OR	16,290	0.647 ± 0.007	0.961 ± 0.002	0.650
One-hot (default)	Fully connected net (FCNN)	84,090	0.73 ± 0.01	0.973 ± 0.001	0.734
Autoencoder	FCNN	12,810	0.731 ± 0.003	0.970 ± 0.001	0.734
UniRep	FCNN	114,930	0.70 ± 0.01	0.963 ± 0.002	0.699
ProtVec	FCNN	3930	0.690 ± 0.003	0.962 ± 0.002	0.683
ESM-1b	FCNN	39,330	0 (model did not converge)	–	–
AbLang	FCNN	23,970	0.715 ± 0.003	0.969 ± 0.0008	0.719
AntiBertY	FCNN	16,290	0.709 ± 0.008	0.968 ± 0.001	0.707
One-hot (default)	Random forest	NA	0.673 ± 0.003	0.956 ± 0.003	0.672
Autoencoder	Random forest	NA	0.7 ± 0.009	0.960 ± 0.003	0.708

This table compares both different encoding types (one-hot, the autoencoder, UniRep, ProtVec etc) and different models (a random forest model, a Bayesian network and a traditional fully connected network) based on classification accuracy.

**Fig. 1:**
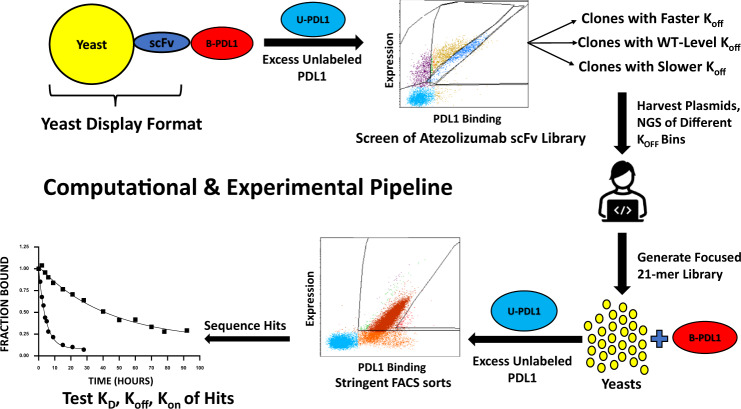
Computational and experimental pipeline. Schematic illustration of the RESP computational and experimental pipeline, U-PD-L1 and B-PD-L1 are unlabeled and biotin-labeled PD-L1, respectively.

**Fig. 2:**
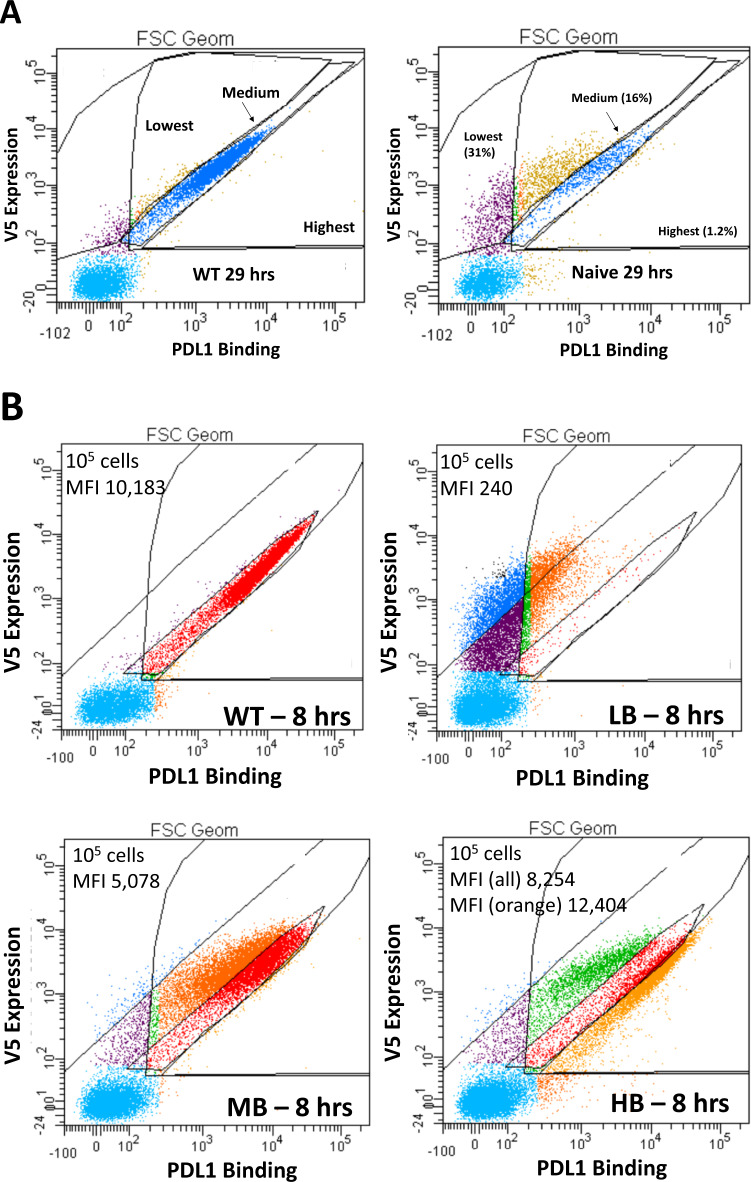
Library sorting by FACS. Sorting of the naive library by FACS and quality assessment of each enriched group by flow cytometry. **A** Sorting of the naive Atezolizumab scFv library on the yeast surface compared to the WT scFv (after 29 h of off-rate competition). The right (highest) gate was set to collect mutants with slower off-rates while the middle (medium (blue cells)) gate was set for WT-level off-rates and the left (lowest (purple)) gate set for faster off-rates. The y-axis represents scFv expression (measured by the level of V5 epitope/AF647) while the x-axis is PD-L1 binding/PE intensity. % values represent each group’s percent of the total number of sorted cells. **B** Testing each sorted population for binding intensity relative to the WT after 8 h of off-rate competition. LB are low-binders (faster off-rates), MB are medium binders (WT off-rates), HB are high binders (slower off-rates). Analyzed 10^5^ cells per group, mean fluorescent intensity (MFI) on the x-axis is given per plot, V5 expression (y-axis) from AF647 intensity while binding (x-axis) in PE intensity.

Second, we construct a yeast surface display library of mutants for a starting antibody sequence (Atezolizumab in this study, where residues were randomly mutated in the antibody heavy chain). The mutant library was incubated with the labeled target antigen (PD-L1 in this study) and screened for mutants with slower dissociation kinetics (slower off-rate – i.e. likely tighter binders) by incubation in the presence of excess unlabeled target antigen. The flow cytometry experiment collects mutants with lower, moderate, and faster off-rates than the WT antibody, thereby binning the population into a series of ranked groups (Figs. 1 and 2). The sequences are then determined by sequencing and their group identities are distinguished using an encoding scheme.

Third, we predict the sequence off-rate by developing a variational Bayesian neural network to model the experimental data through ordinal regression. This affinity model takes as input the encoded sequences from the yeast surface display library and tries to predict the likelihood that a given sequence has a slow off-rate (i.e. is a tight binder). The variational Bayesian architecture provides strong regularization that minimizes the risk of overfitting and estimates the model’s uncertainty on each prediction. By using ordinal regression, we mapped each sequence to a one-dimensional latent score that indicates the model’s level of confidence to which sorting group the sequence belongs. This approach naturally takes into account the ranked ordering of the groups and is a useful approach as we have demonstrated previously for protein engineering problems^40^. The affinity model should be retrained for a new antigen or a different starting antibody sequence, while its architecture can be kept unchanged.

**Fig. 3:**
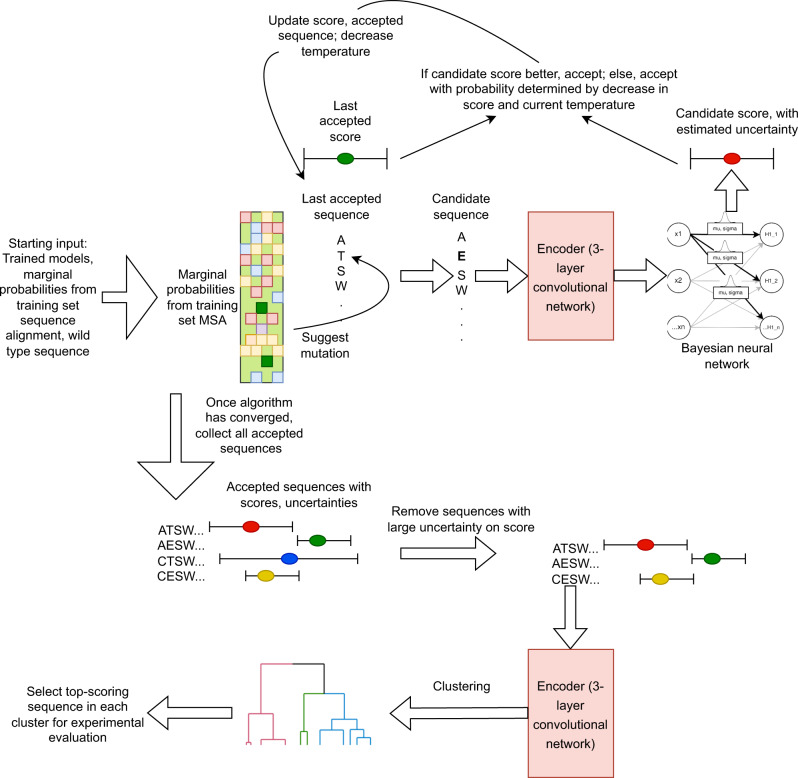
Overview of the RESP pipeline. In silico directed evolution using approximate Bayesian inference and learned embeddings for efficient candidate sequence evaluation.

Finally, we have modified the simulated annealing algorithm to develop an in silico directed evolution algorithm that harnesses the three previous pieces of the pipeline to efficiently explore the sequence space surrounding the training set and identify sequences likely to exhibit off-rates significantly lower than that of the parent sequence. The resulting analysis pipeline is illustrated in Fig. 3. This component provides an efficient approach for exploring the sequence space and can be performed using a Bayesian neural network trained for any antibody-antigen pairing of interest.

In this study, we have applied the RESP pipeline to improve the binding affinity of Atezolizumab, an antibody targeting PD-L1. We selected 21 candidate sequences with predicted slower off-rates than the WT sequence and experimentally showed that one of them has an off-rate about 10-fold slower and *K*_D_ 17-fold tighter than the WT Atezolizumab scFv.

Furthermore, we have evaluated the core computational components of RESP on data acquired by Mason et al.^11^ and demonstrated that our models showed the same accuracy as the CNN model in the original study while providing additional useful uncertainty information.

We now discuss each component of the pipeline in more detail.

### Encoding antibody sequences using an autoencoder model

**Fig. 4:**
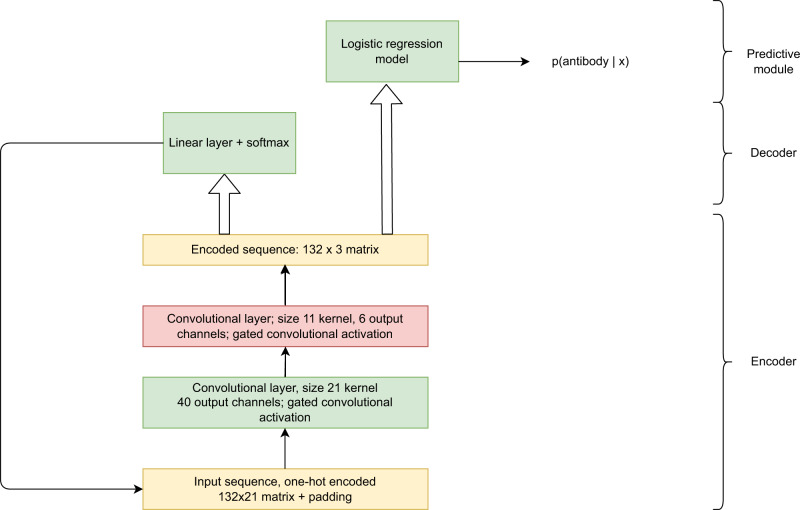
The autoencoder architecture. The structure of the task-adapted autoencoder. The encoder module generates a learned representation for each input sequence; the decoder module tries to reconstruct the input sequence, while the classifier generates a binary prediction for decoy vs. human B-cell receptor in its training set.

We first developed an autoencoder model trained to represent antibody sequences and distinguish true antibody sequences from other closely related sequences. We reasoned that the requirement to distinguish antibodies from closely related sequences might force the encoder to embed information about typical preferences at specific positions into its learned representation. To this end, we have built a convolutional autoencoder consisting of three main modules or components, illustrated in Fig. 4 (for details, see “Methods”) and compared its performance with one-hot encoding, UniRep^33^, ESM-1b^35^, ProtVec^34^, AntiBertY^31^ and AbLang^32^. This component of the pipeline is general and does not need to be retrained for a new antigen or wild type.

We drew our training set for the autoencoder from the cAb-Rep database^41^, which contains sequenced B-cell receptor repertoires for 121 human donors representing true antibody sequences. Specifically, we filtered the high-depth repertoire dataset using the ANARCI software package^42^ (see “Methods” for details) to remove incomplete sequences and numbered the surviving sequences using the Chothia numbering scheme, resulting in a dataset of 2,725,492 sequences. We then augmented this dataset with an equal number of decoys, generated by making a copy of each true antibody sequence and randomly modifying it at 7 positions. This number was chosen to strike a balance: increasing the number of mutations makes it more unlikely that any mutant will coincide with actual human B-cell receptor sequences occurring in nature. Too many mutations however makes it too easy for the classifier to distinguish the decoys from the rest, so that the encoder unit will no longer be forced to learn an information-rich representation of the input. We experimented with different numbers and 7 mutations provided a good balance.

**Fig. 5:**
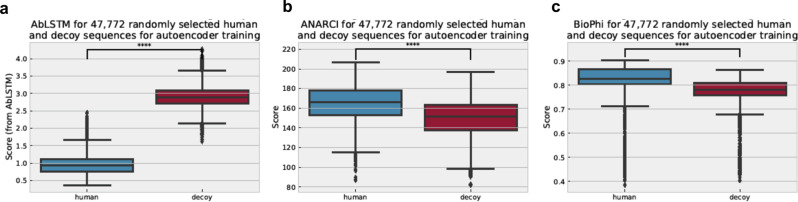
Scoring the human-ness of decoys and original sequences from the autoencoder training set. We randomly sampled 47,772 sequences from the autoencoder training set (half decoy, half human). We then score these for human-ness using **a** the AbLSTM model, **b** the ANARCI tool and **c** the BioPhi model from the literature. In all three cases, the model’s score for decoys is significantly different from that for non-decoys, and the decoys are less human than the original sequences. In all three cases, using the two-sided Mann–Whitney *U* test as implemented in Python’s Scipy library version 1.5.4, the calculated *p*-value is 0.0 (meaning that it is approximately 0 given floating point error). The following conventions apply for each boxplot. The upper and lower bounds of the box are the 25th and 75th percentile of the data, and the whiskers are drawn at 1.5× the interquartile range (the distance from the 25th percentile to the 75th percentile). The center is drawn at the median of the data, and the “notch” represents the 95% confidence interval on the median (as determined by nonparametric bootstrap). The diamonds represent “flier” points which lie outside 1.5× the interquartile range. Four asterisks indicates the *p*-value is <0.0001. Source data are provided as a source data file.

To ensure that the decoys were in fact different from typical human antibody sequences, we scored a random sample of approximately 50,000 original sequences and decoys with three different models of BioPhi^43^, AbLSTM^44^, and ANARCI^42^ (see Fig. 5). Using any of the three models/tools, the decoys exhibit significantly different human-ness scores from the original sequences (two-sided Mann-Whitney U test. For all three rating methods, using Python 3.9, Scipy 1.5.4 and Pingouin 0.5.2, both Scipy and Pingouin indicate the *p*-value is too small to calculate reliably given floating point error on double precision arithmetic and return ≈ 0.0). The decoys are therefore clearly rated by the models as less human, demonstrating that they are suitable to serve as decoys.

The autoencoder was trained on the full one-hot encoded cAb-Rep plus decoy dataset with a test set of 200,000 sequences set aside for 8 epochs at which point the training loss had converged. The reconstruction accuracy on the test set was > 99.99%, while the prediction accuracy for the B-cell receptor vs mutant task was 97.4%. These results suggest the autoencoder can compress the input sequence while retaining all the information needed to reconstruct or classify it. We will assess the performance of the autoencoder as an input for models to predict binding affinity below under the Atezolizumab modeling section.

### Generation and screening of the Atezolizumab scFv heavy chain library for improved off-rates

To use the RESP pipeline to develop antibodies against a target antigen, we must first generate training data specific to that target antigen, so that we can train a model to recognize the types of sequences that will bind it. In this study, our test case is the antigen PD-L1, and our starting point is the single-chain fragment variable (scFv) heavy chain of the Atezolizumab antibody against PD-L1, hereafter termed the wild-type (WT).

We first tested the WT Atezolizumab scFv for functionality in the yeast display format by testing binding to human PD-L1 (Supplementary Fig. 1), and demonstrated robust binding with 6.1 nM of antigen present. The variable heavy (VH) domain of Atezolizumab was chosen for mutagenesis because the structures of the Fab of Atezolizumab bound to PD-L1 show that the heavy chain is primarily involved with the binding interaction^45,46^ (PDB codes 5XXY, 5X8L). Also, mutating only the heavy chain facilitates deep sequencing because of the shorter region needed to be read by MiSeq (in this case, a region of approximately 316 bp). Error-prone PCR^47,48^ of the heavy chain region was used to randomly generate mutations of the gene, followed by transformation into EBY100 yeast to create a library of up to 78 million Atezolizumab scFv variants. Because the binding interaction and off-rate between the WT scFv and PD-L1 was very strong/slow (*K*_D_ = 1.75 nM, *k*_off_ = 1.56 × 10−^4^ s^−1^)^49^, yeast display is better suited under these conditions at decreasing the off-rate rather than directly improving the *K*_D_ because of the large volumes necessary to maintain a large molar excess of antigen over the scFv on the yeast surface along with the very long incubation times needed to screen for improved *K*_D_ when the off-rate is already very slow^50^. Using the WT scFv as a control during sorting, variants with faster, WT-level, and slower off-rates were isolated from FACS sorts (Fig. 2A) and had their plasmids harvested and subjected to multiple rounds of PCR to barcode each group for MiSeq Nano PE250. Notably, the clones selected to have slower off-rates seemed to split into two populations, one more intense than the WT (orange cells, suggesting slower off-rates) and less intense than the WT (green cells, suggesting faster off-rates) (Fig. 2B).

The Atezolizumab dataset consists of sequences derived from mutants in 3 different bins of decreasing off-rates: RH01, RH02 and RH03. RH01 contains weak binders with a faster off-rate, RH02 contains moderate binders with an off-rate similar to the wild type, and RH03 contains stronger binders with slower off-rates. After filtering for quality, 92,553 unique sequences (550,215 total sequences, since a sequence can appear more than once in a bin or more than once in different bins) were identified, among which 15,004 sequences exhibiting mutations outside the region of the protein targeted for randomization were discarded. There were 15,070, 26,122 and 34,439 unique sequences in RH03, RH02 and RH01, respectively (Supplementary Table 1).

Each sequence was assigned to the category in which it occurred with the greatest frequency and assigned a weight given by its frequency in the assigned category plus one divided by the total frequency plus three. If the frequency for a given sequence was equal in two categories, it was discarded since it could not be unambiguously assigned. As a result of these filters, 75,631 unique sequences remained. The weight of each sequence corresponds to the posterior probability that it belongs to a given category using a multinomial likelihood and uniform Dirichlet prior (for more details, see “Methods”). This weighting is important since it enables a model to distinguish between sequences that can and cannot be reliably assigned to a single category. A sequence that appears 5 times in both RH02 and RH03, for example, is clearly less likely to be a strong binder than a sequence that occurs 5 times in RH03 only.

### Affinity modeling of the sorted sequences

The next component of the pipeline is an affinity model which takes as input a representation of a candidate sequence and predicts its sort category (RH01, RH02 or RH03) to which the sequence should belong. This component requires experimental data specific to the antigen of interest and will need to be retrained with fresh experimental data if a new antigen of interest is selected.

Unlike most classifiers for protein engineering, the affinity model is trained to perform ordinal regression and the last layer of the network outputs a latent score value. A traditional classification model treats the categories as nominal–they have no particular ordering–and thus the model does not learn any way to rank the categories. This is clearly inappropriate for ordinal sort data. Ordinal regression solves this problem by imposing an ordering on the categories and by using a latent score to determine into which category the sequence should fall. This approach provides a straightforward means to rank sequences and select them for experimental evaluation^40^. In past experiments^40^, we demonstrated that given protein data with >3 binding categories, ordinal regression provides improved performance for correctly predicting which sequences will occur in future, more stringent sorts, and in particular outperforms the sequence ranking approach suggested by Liu et al.^10^ Those experiments are difficult to reproduce on this data since in this case we have only 3 binding categories, but given our past results we use ordinal regression here as well.

The affinity model output score quantifies the extent of the model’s belief that the sequence is likely to be a strong binder–higher scores indicate the model is more certain the sequence should belong to a more stringent sort. The score is next added to *M − 1* learned threshold values for *M* categories, followed by application of the sigmoid to generate an output vector of *M − 1* probability values. Each element *i* of this output vector is the model-assigned probability that the input sequence belongs to a binding category more stringent than *i*.

Briefly (for details see “Methods”) our affinity model employs an architecture similar to the Bayes by Backprop algorithm^51^, with the difference that our model is adapted to perform ordinal regression. A traditional fully connected neural network learns a specific value for each weight and bias term in each hidden layer during training. A Bayesian neural network, by contrast, treats each weight as a (usually Gaussian) distribution and learns the parameters for each weight’s distribution during training. Instead of fitting using maximum likelihood as is typical for neural networks, the Bayesian architecture is fitted by approximating the posterior probability distribution using a variational method. By sampling over the weight distributions repeatedly, we can estimate the uncertainty in a prediction–or if preferred use the mean of each weight’s Gaussian distribution to generate a point estimate prediction. In addition to generating an estimate of uncertainty, the Bayesian neural network also provides strong regularization on the model parameters by penalizing deviations from the prior.

The uncertainty information provided by the model can clearly be used in one of two ways. Predictions with high associated uncertainty correspond to sequences that lie in relatively unexplored regions of the input space. In practice, only predictions with relatively small associated uncertainty should be selected for experimental evaluation considering the cost and time, and that is the strategy we pursue here.

We compared the results generated by a vanilla fully connected neural network trained to perform simple classification with a Bayesian net trained to perform ordinal regression across various encoding types (Table 1). Note that both neural networks compared have the same number of hidden layers and weights. Although the non-Bayesian network offers a modest improvement in performance, the Bayesian network offers additional information about the uncertainty associated with a given prediction that is crucial for analyzing the sort data, since it enables us to gauge the relative reliability of the model’s predictions for candidate sequences.

The affinity model can use one-hot encoded sequences as input or another representation. To determine whether the representation generated by the autoencoder is useful as an encoding for the affinity model, the Atezolizumab sequences were encoded using (1) the fully trained autoencoder, (2) one-hot encoding, (3) the ProtVec encoding^34^, (4) the UniRep embedding^33^, (5) the ESM-1b encoding^35^, (6) the AbLang embedding^32^ and (7) the AntiBERTy^31^ embedding. The encoded datasets were split into a training (80%) and test (20%) portion, and a fivefold cross validation was performed on the training portion for each encoding type, using a Bayesian neural network trained to perform ordinal regression (Table 1).

While different encodings may well prove preferable for specific tasks, the autoencoder is the only representation equivalent or superior to one-hot encoding of antibodies for any model type (Table 1). It offers equivalent performance despite a significant reduction in model size and computational expense compared to the one-hot encoding. Note that the autoencoder has roughly 20,000 parameters, while the FAIR-ESM model has 750 million parameters and the AntiBERTy model has 26 million.

The autoencoder therefore provides at least two concrete benefits. First, it reduces model size and complexity significantly compared with simple one-hot encoding, and it is substantially cheaper than language models, since it has a small fraction of the number of parameters. Second, it provides a real-valued representation of each sequence that can be used to cluster sequences or determine similarity between them in later stages of the pipeline. Given these advantages, we prefer the autoencoder to one-hot encoding even though in this case they achieve similar performance.

### In silico directed evolution to select the most promising candidates

We encoded the Atezolizumab dataset using the autoencoder and then trained the Bayesian neural network (the affinity model) on the full dataset for off-rates for 30 epochs. At this point, our pipeline uses the trained affinity model to search for the sequence space surrounding the training set to generate new candidate antibody sequences not present in the training set.

At this stage it is desirable to reduce the size of the search space. If we mutate a 118 amino acid sequence in silico, the search space to be covered is impossibly vast. We prefer therefore to focus on a smaller subset of positions that contribute significantly to binding affinity—those most frequently mutated positions in the top-scoring sequences, i.e. the most promising sequences considered by the model. For this dataset, we found that, using the top 500, 1000, 2000 or 4000 highest-scoring sequences to select the top ten most frequently mutated positions, we retrieved the same set of positions that are used in the subsequent search steps. This step in the pipeline is flexible and positions can be selected using other criteria (e.g. only frequently mutated positions present in CDRs).

In the following search steps (for details, see “Methods”), on each iteration the model randomly selects a position in the wild-type sequence and randomly mutates it to any amino acid. Note that all mutants generated through this procedure have the same length as the wild-type sequence. The probability of selecting any specific amino acid at a given position is given by the frequency of that amino acid in the training set plus one, divided by the total number of sequences in the training set plus 20. The new candidate sequence is scored by encoding it using the encoder and scoring it using the trained affinity model. The score is assessed using the classic simulated annealing criterion, whereby the candidate is accepted with a probability determined by its score, the score of the last accepted candidate and the temperature. The temperature begins at a high value to allow exploration of a large sequence space and decreases on each iteration, so that the probability of accepting a proposed sequence without an improved score decreases.

**Fig. 6:**
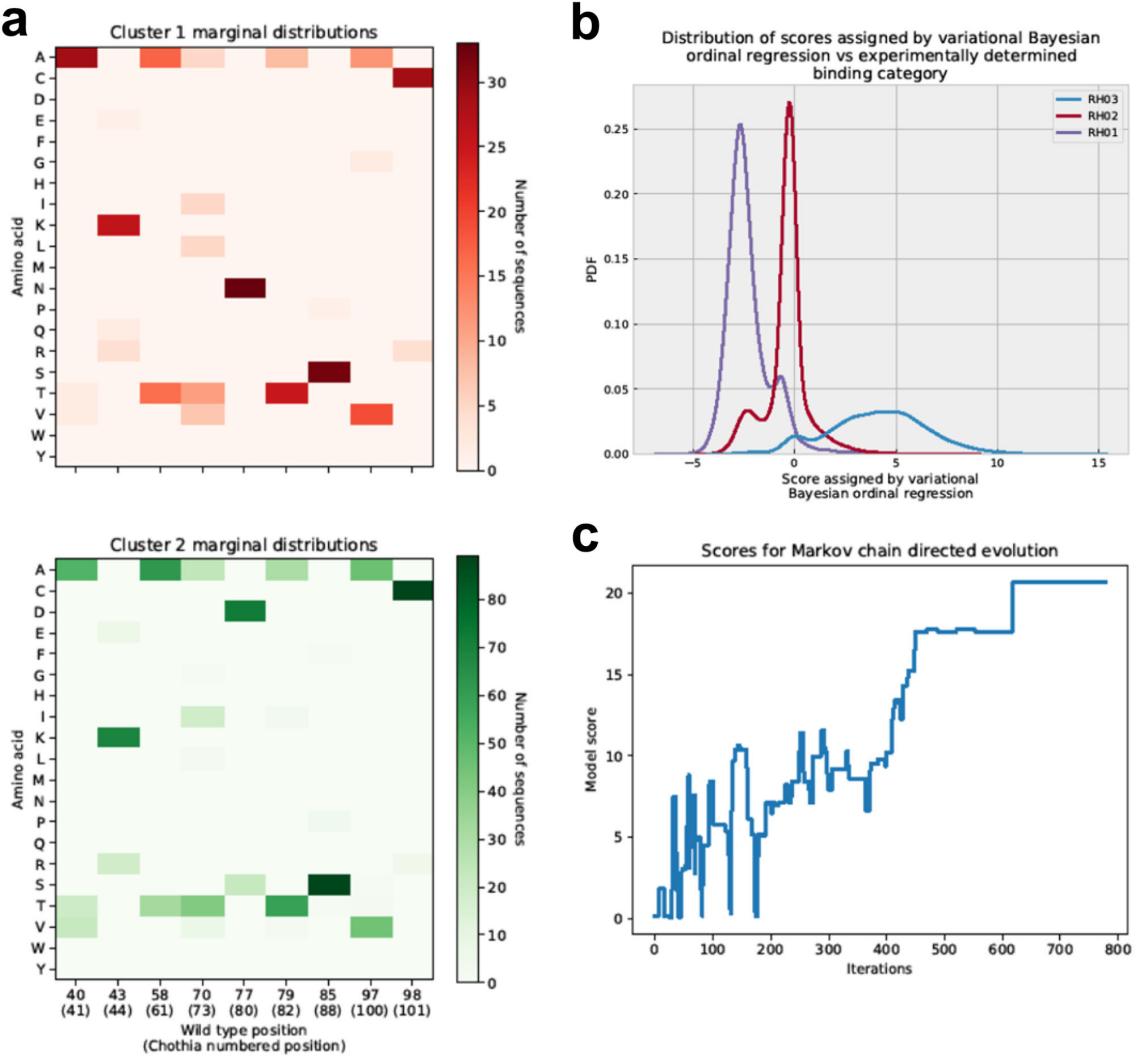
Analysis of pipeline results. **a** The per-position marginal distributions in the two main clusters. Each main cluster has several subclusters as apparent from the marginal distribution. Only positions mutated in either cluster are displayed. Each position is numbered first using numbering from the wild-type sequence and then in parenthesis using Chothia numbering. **b** illustrates the distribution of scores assigned to sequences in each binding category by the variational Bayesian model, which is designed to perform ordinal regression (classification on ranked categories). The experimental data does not directly measure off-rates but rather classifies sequences into three bins: RH01 (high off rate), RH02 (moderate off rate), RH03 (low off rate). The higher the score assigned by the ordinal regression model, the more confident the model that the sequence has a low off rate, while lower scores indicate greater confidence in a high off rate. **c** The accepted scores vs iteration for a typical simulated annealing chain. The algorithm initially explores sequence space impartially and as the temperature is reduced is gradually forced to focus on the most promising regions it has found thus far. Source data are provided as a source data file for all panels.

Since the average sequence in the training set contains just 7 mutations and the average high-scoring sequence contains even fewer (3 mutations on average), at most positions the most common amino acid is the one present in the wild type. Consequently, the algorithm will tend to heavily sample sequences that are similar to the wild type. Given the stochastic nature of the algorithm, however, it can explore combinations not present in the training set and thereby enable us to find new high-scoring sequences. Figure 6 illustrates how the best achieved score to date evolves over the course of this optimization.

We ran 10 simulated annealing chains; all converged in under 1000 iterations. The accepted candidates from each chain with scores > 90th percentile were harvested and duplicates were removed. We evaluated the uncertainty on the scores by sampling 1000x from the Bayesian neural net for each sequence. The top 50% of sequences with the largest standard deviation on assigned scores were removed, since we are least confident about these predictions. The 50% threshold here is arbitrary and this process yielded 127 sequences.

We clustered these using median hierarchical clustering (the resulting dendrogram is included in the Supplementary Information as Supplementary Fig. 11). The results clearly suggest the selected sequences can be divided into two main subgroups. The marginal distributions (see Fig. 6) indicate the two major groups differ primarily at position 77 but also contain subgroups with some other interesting differences. Most selected sequences, for example, exhibit a R98C mutation, but a subset of cluster 1 is unchanged from the wild type at that position.

We cut the tree at a lower height to yield 11 subclusters and selected the two highest-scoring sequences (or one if only one sequence was present) in each cluster to yield 21 final candidates. This threshold was selected to yield a manageable number of sequences for experimental evaluation.

**Fig. 7:**
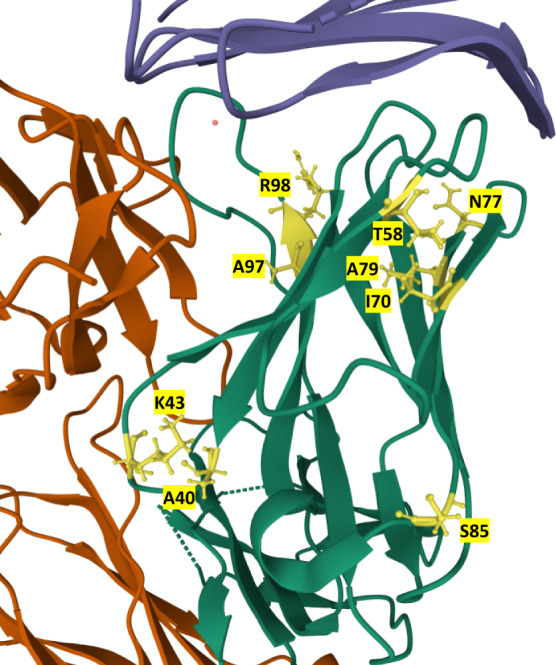
The locations of mutations in the PD-L1-Atezolizumab complex. Location of mutations in the 21 mutants in the structure of the Atezolizumab heavy chain. Mutated residues are labeled and colored in yellow in the heavy chain (green). PD-L1 is colored blue while the light chain is orange. Structure from RCSB Protein Data Bank (5XXY) in Mol* Viewer^45, 71^.

Examination of the mutations within the 21 sequences revealed mutations at residues A40, K43, T58, I70, N77, A79, S85, A97, and R98 (in various combinations, see Supplementary Table 3 and Fig. 7). Examination of the existing structures of the Atezolizumab Fab and PD-L1 (PDB codes 5XXY and 5X8L) reveals that none of the mutated residues contact PD-L1 in the WT sequence (Fig. 7). This suggests these mutations improve the binding affinity through alterations of the heavy chain conformation rather than directly improving binding contacts. Another possibility is that these mutations stabilize the conformation rather than altering it. Ruffolo et al. demonstrate, for example, that in some cases stabilizing a specific antibody conformation can be beneficial for affinity^52^.

To explore these possibilities, we generated predicted structures for the top-scoring mutants from the 21 sequences using IgFold^53^ without antigen. We note that the mutations present in high-scoring sequences shift the conformation of both antigen-contacting regions of the protein, especially the CDR H3. In the Supplementary, Supplementary Figs. 8 and 9 show the conformational change of R98C (a mutation present in many of the top-scoring sequences) and its surrounding residues as well as and the formation of an apparent hydrophobic pocket in the top mutant compared to the wild type. Note that the mutations we describe are not in direct contact with the antigen. These predicted structures suggest that the mutations selected by our pipeline function through changing the conformation of the contact regions rather than by directly forming new contacts with the antigen by themselves.

We also use the trained affinity model to computationally assess the importance of individual mutations. We score (A) how much a mutation would contribute to binding when only itself is introduced individually to the WT without other mutations and (B) how much binding affinity would change if a single mutation is removed from a beneficial mutation combination. The full results are presented in Supplementary Tables 4, 5 and summarized here. The results do suggest that some mutations may be more important than others, but also suggest that 12 of the 19 mutations introduced are predicted to be beneficial even in isolation, i.e. to increase the likelihood that the sequence in question will be a tight binder. The remainder are very slightly detrimental in isolation or cause no change, although they are predicted to have beneficial impact in certain contexts. A[79]I or A[79]T, R[98]C, I[70]A, A[97]V and T[58]A are predicted by the model to be mutations with large beneficial impacts even in isolation.

Interestingly, the model is able to predict synergy between individual mutations. For example, K43Q is predicted to have an almost negligible beneficial impact if introduced into the wild type in isolation, but is predicted to have a much larger negative impact on the score if it is removed from the mutant K[43]Q,A[79]T,A[97]V,R[98]C. A[97]T is very slightly detrimental in isolation, but is predicted to be beneficial for A[40]T,K[43]E,T[58]A,N[77]D,A[79]T,A[97]T,R[98]C. While A[79]T, for example, is predicted to be beneficial no matter in what context it is introduced, the size of that impact on the score varies as much as twofold depending on the other mutations present.

### Validation of the predicted tight-binding antibodies

The 21 mutants (all in the heavy chain) generated by the model were purchased as geneblocks and fused with the WT light chain sequence by PCR, followed by transformation into EBY100 yeasts. The small library of 21 mutants was screened in a similar manner as the naive library, except a longer competition time was used in the final screen (39 h) in an attempt to separate the mutant with the slowest off-rate from the rest of the 21 mutants (Supplementary Fig. 2). This process did not result in a clearly separated mutant and sequencing of random clones after the most stringent sort showed that out of 17 random sequences, 12 separate mutants (occuring in similar frequencies) were found to be present. This suggests that a significant proportion of the 21-member pool of mutants had significantly slower off-rates than the WT (which exhibited significantly reduced fluorescence when compared with the pool of mutants in the final, most stringent sort (Supplementary Fig. 2, lower panels). It also appeared that essentially all of the 21 mutants selected by the model had slower off-rates than the WT upon examination of the original/unsorted 21-member library after 8-hours of off-rate competition at RT (Supplementary Fig. 2 top panels).

**Fig. 8:**
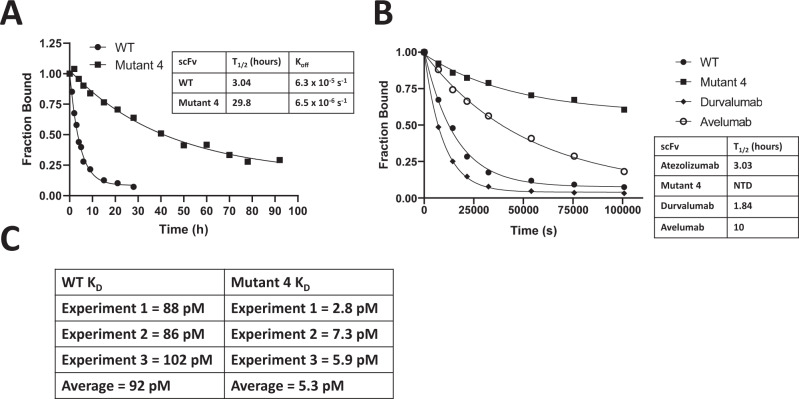
Determination of the off-rate and *K*_D_ of mutant 4. Experimental validation of the *K*_off_ and *K*_D_ on the yeast surface. **A** WT vs. Mutant 4 scFv dissociation after 92 h at RT on the yeast surface, T_1/2_ is half-life. **B** Comparison of WT Atezolizumab, Mutant 4, Durvalumab, and Avelumab scFv dissociation over 28 h at RT on the yeast surface. **C** Binding affinity (*K*_D_) measurements determined on the yeast surface between scFv and PD-L1 (3 independent measurements, also see Supplementary Fig. 7)). Source data are provided as a source data file for this figure.

From the various mutants selected from the 21-member library after the most stringent sort, we selected the I70A/A79T/A97V mutant (named Mutant 4, see Supplementary Table 2 for sequence) for further characterization. This mutant, unlike the other mutants sequenced after the stringent sort, did not have cysteine introduced into its sequence so was more appealing because additional disulfide bonds would not be formed by its mutations. In order to characterize the improvement in off-rate/binding towards PD-L1 of the isolated mutant, we determined the *K*_D_ and *k*_off_ values of both the WT and Mutant 4 scFv towards PD-L1 using the yeast display format^48,54^. First, we determined the apparent off-rates at RT on the yeast surface (Fig. 8A) and found that Mutant 4 has a *k*_off_ approximately 10-fold slower than WT Atezolizumab (6.3 × 10^−5^ s^−1^, half-life 3.04 hrs for the WT vs. 6.5 × 10^−6^ s^−1^, half-life 29.8 h for Mutant 4). We then compared the *k*_off_ values of Mutant 4 to the two other FDA-approved anti-PD-L1 mAbs (Durvalumab and Avelumab, converted to the scFv format on the yeast surface) (Fig. 8B). Mutant 4 displayed a substantially slower off-rate compared to both WT Atezolizumab and Durvalumab and a noticeably slower value than Avelumab. The order of off-rates of the FDA-approved mAb PD-L1 scFvs (Durvalumab <Atezolizumab <Avelumab) is consistent with a previous study of these scFv values by a surface plasmon resonance (SPR) binding assay^49^.

Next, we attempted to determine the binding affinity (*K*_D_) values of the WT and Mutant 4 scFv by titrating PD-L1 against each scFv on the yeast surface. The Mutant 4 *K*_D_ was about 17-fold tighter (on average) than the WT (5.3 pM vs. 92 pM, Fig. 8C). It should be noted that determining the *K*_D_ on the yeast surface at such low concentrations of antigen is difficult, given the excessive volumes necessary to maintain a significant molar excess of antigen to scFv on the yeast surface at lower antigen concentrations^48^, which also makes performing replicates for each datapoint difficult. However, three separate *K*_D_ measurements gave significant (>10-fold) improvements in the binding affinity, providing confidence in the figures listed in Fig. 8C.

In order to assess if the improved binding affinity and off-rate observed on the yeast surface can be repeated after purifying each scFv and reversing the orientation of the binding assay (instead of scFv fused to the yeast surface and binding to soluble PD-L1, PD-L1 is immobilized and allowed to bind to soluble scFv), we performed a BLI (Bio-Layer Interferometry) binding assay and found that Mutant 4 has a much slower off rate than the WT, consistent with the yeast surface display results. The dissociation of the mutant from PD-L1 in this assay was minimal even after 2 h at RT while the WT was more significant over about a 60 min dissociation time. However, BLI gave two *K*_D_ values for the WT (219pM for the major species and 5.93 nM for the minor species) while the Mutant 4 *K*_D_ values were immeasurable because of its very slow off-rate, leading to an inability to fit/determine the kinetic constant values (see Supplementary Fig. 3). We suspected the minor species was caused by a minor population of partially unfolded scFv in the protein preparation. This observation prompted us to examine whether the scFv and PD-L1 were monomeric or formed oligomers, so we tested this by mass photometry (a method that can image oligomeric distributions of a protein in solution^55^). Imaging PD-L1 (residues Met1-T239, expected MW 35-38 kDa, range in MW due to glycosylation) revealed a predominantly monomeric protein at the concentrations tested (7.4 nM, 18.5 nM, and 74 nM) (Supplementary Fig. 4), consistent with previous studies of human PD-L1 which found it to be monomeric^56,57^. This data supports that the *K*_D_ measured on the yeast surface is between monomeric PD-L1 and scFv immobilized to the yeast surface. Imaging of the WT or Mutant 4 scFv in solution revealed a mixed population of monomer, dimer, trimer, and possibly higher MW oligomers at 100 nM each scFv. The WT and Mutant 4 scFv were purified twice (using the same exact method) and tested twice by mass photometry. For the WT scFv, in both preparations it was a mixture of monomer and dimer in solution while for an unknown reason the mutant was either predominantly monomeric (1st batch) or a mixture of monomer/dimer/trimer (batch 2) (Supplementary Fig. 4). Furthermore, we noticed that the mutant scFv seemed more prone to aggregation than the WT scFv. Taken together, the oligomerization and aggregation of scFv in solution makes binding assays like SPR (surface plasmon resonance) or BLI, where scFv is in solution and PD-L1 is immobilized, inappropriate for *K*_D_ determination because of the difficulty of knowing the soluble monomeric concentration of scFv.

### Evaluating the generality of RESP on an additional independent dataset

To ensure the generality of RESP, we next tested it on the data from the Mason et al. study^11^. Starting from a nonbinding mutant of trastuzumab, they successfully recovered antibodies with similar affinity to the original wild-type trastuzumab. Using the same training and test sets, we encoded their sequence data using our autoencoder and trained a variational Bayesian network with the same structure we used for our data to predict binding and nonbinding status.

Our model achieves the same accuracy as theirs (Matthews correlation coefficient 0.68, AUC-ROC 0.91) for their test set and correctly predicts the sequences they experimentally tested to be binders. (For further details, refer to Supplementary Fig. 10). Our model likewise assigns high scores to most of these sequences. Trastuzumab, for example, is assigned a higher score than 94% of the sequences in the training set, indicating a high confidence that it is a tight binder. The distribution of scores for the training set and for the experimentally evaluated sequences appear in Supplementary Fig. 10.

Unlike the Mason et al. pipeline, however, ours provides additional uncertainty information not available from their model. Our affinity model’s uncertainty about test set predictions which turn out to be incorrect is significantly higher than its uncertainty about test set predictions which *are* correct (*p* < 1e−23, two-sided Mann–Whitney *U* test). Consequently, we can use uncertainty to assist in determining whether a prediction is likely to be reliable, which is not possible with the pipeline developed by Mason et al., and we do not need to sacrifice accuracy to obtain this advantage.

## Discussion

We present here a pipeline for efficiently developing antibodies with the following innovations. First, we develop a simple learned encoding specific to antibodies. Our encoding contains not only the information in the original sequence in easily recoverable form but also additional encoded information describing key sequence features that differentiate human antibodies from the surrounding sequence space. We demonstrate that when training a model on antigen-specific experimental data to capture trends in binding affinity, the same model is more accurate if the input sequences are encoded using our autoencoder-generated representation than if the input sequences are instead encoded using popular state-of-the-art encodings like UniRep, ESM-1b, AntiBertY and AbLang. Remarkably, for this particular task, using all of the models we consider, UniRep, ESM-1b, AntiBertY and AbLang exhibit performance inferior to one-hot encoding. This is consistent with results reported by Makowski et al, who found that UniRep or physicochemical properties did not improve performance for antibody affinity prediction compared with simple one-hot encoding^58^.

Next, we fit our training set using models designed to provide both straightforward and easy to interpret sequence ranking coupled with quantitation of uncertainty. We show the distribution of sequence reads across categories can be incorporated into model fitting as a datapoint weight in a principled and straightforward way. Our Bayesian ordinal regression model yields an estimate of the predictive posterior, thereby providing additional information not available from traditional deep learning classifiers, whose predicted probability distribution across categories does not indicate the reliability of a given prediction.

Finally, we design an algorithm to explore the sequence space spanned by the training set. By estimating the reliability of each prediction and by restricting our search to the space spanned by the training set, we minimize the time and expense wasted on evaluating poor candidate sequences.

We experimentally validated the power of this pipeline. By training our model on a single large library, we were able to select a mutant with an off-rate/binding affinity improvement of 10-fold/17-fold. This is consistent with our past results for protein engineering of CBX1, where we demonstrated a similar strategy yielded improvement in binding affinity equivalent to that achieved by a much lengthier directed evolution process^40^. We note in passing that the Mutant 4 which we identified could be useful in cancer therapy as an scFv as was a previously reported high-affinity anti-PD-L1 protein (a mutant form of PD-1, which bound PD-L1 with a *K*_D_ of 110 pM^59^). Like PD-1, the Mutant 4 scFv is significantly smaller than a monoclonal antibody (30 kDa vs. 150 kDa) and so could possibly be more effective at tumor penetration^59,60^.

We observe that this pipeline has several important advantages over purely experimental approaches. Phage and yeast display only permit selection of a small population, not of single clones, so that additional experiments (such as ELISA or yeast *K*_D_ measurements) are needed to assess the clones having the tightest binding affinity. Only strong binders present in the original library can be identified via these techniques, so that often very large libraries and/or multiple libraries are used to maximize coverage of sequence space. It often happens that the best binders identified through this process still do not possess sufficiently strong affinity, so that the desired affinity must often be achieved through so-called affinity maturation. In this process, random mutations are introduced at selected sites and the resulting focused library undergoes further rounds of screening and experimental evaluation. Notably, the antibodies generated through this process are not guaranteed to possess other desirable characteristics like solubility or stability.

Take for example the process by which the Atezolizumab antibody itself was originally discovered (as described in US Patent US8217149B2). Four rounds of panning on a phage display library screened against the PD-L1 target were first used to retrieve 96 enriched clones. Two sets of positions were then selected to construct two further libraries for affinity maturation to improve binding. These in turn were used to conduct one plate sort followed by five or six rounds of solution sorting. Finally, enriched clones from the last sort were run through high-throughput ELISA screening to find the best candidate. It is immediately obvious that this procedure, while reliable, is expensive in time and cost. We note that it is very common that antibody engineering requires construction of multiple libraries and high-throughput ELISA of thousands of clones; for a couple of other examples involving antibodies now in clinical trials, see US Patent 20180086848A1 and 8313746B2 among others.

In our approach, we were able to select a tight-binding antibody after constructing only a single large library followed by FACS-based sorting for off-rate and binning. This approach does not require any high-throughput *K*_D_ determinations or subsequent focused library screens. We thereby eliminate the time needed to construct multiple libraries, which is considerable, and the time and expense needed for high-throughput ELISA screens/*K*_D_ measurements. We replace this with some computational steps which are easy to implement and run quickly on a single computer equipped with a GPU. The Bayesian neural network will need to be retrained any time a new antigen is selected on data specific to that antigen and acquired as we describe.The autoencoder, by contrast, can of course be reused and need not be retrained. Moreover, our approach identified tight binders not present in the original library, unlike traditional phage and yeast display, in which only sequences present in the library can be screened.

It is important to note that our approach can easily be modified to introduce in silico screens for stability, solubility and other desired properties, unlike purely experimental techniques which cannot easily optimize for these other properties simultaneously. It is straightforward to add additional filters to the search step of the pipeline – i.e. to reject candidates suggested by the modified simulated annealing algorithm if predicted solubility or immunogenicity is poor. In a purely experimental approach, by contrast, these properties must be optimized separately at considerable additional expense. The ability to achieve improved affinity while easily introducing additional filters as desired is a key advantage for a machine learning-assisted approach.

There are other strategies for computer-assisted antibody design described in recent literature. We find our approach compares favorably and offers several advantages. For example, Mason et al.^11^ achieve a 3× improvement in affinity over the wild-type trastuzumab, and most of the mutants selected by their algorithm as promising in fact show weaker affinity for the target, whereas we achieve a 17-fold improvement in affinity. Mason et al. performed multiple rounds of mutagenesis and library generation, including a step where positions for further mutagenesis were selected using rational design based on data from a previous selection step. They require this additional effort in order to constrain the search space, which we are able to constrain in silico using our modified simulated annealing algorithm. Unlike the CNN they propose, our Bayesian neural network provides uncertainty estimates correlated with the accuracy of a prediction so that predictions which are likely to be unreliable can be eliminated.

Warszawski et al.^61^ describe a rational design approach (as opposed to a machine learning-based approach). Their rational design component, however, can only be as accurate as the predictions of the Rosetta modeling software, which results in a low rate of correct predictions, nor is it possible to determine which predictions are most likely to be reliable. They sought for example to improve on the binding of an antibody called G6 to its target, VEGF, using a computational search procedure requiring approximately 250 cpu-days, which is orders of magnitude more expensive than the computational procedure we adopt here. Of the mutants selected by this AbLift procedure for experimental evaluation, 60% were worse than the wild type, and most of the remainder were only comparable. Only one of the designs suggested by their approach actually improved the *K*_D_, resulting in a fivefold improvement, which is a smaller improvement than we achieve. It is worth noting that most or all the 21 mutants selected by our model showed slower off-rates (thus likely higher binding affinity) than the WT.

Khan et al.^62^ use a Gaussian process to model trends in binding affinity as a function of input sequence. These authors did not experimentally validate their approach, instead using affinity predictions from the Absolut! software^63^ to determine whether a sequence was a strong binder; the Absolut! Software is itself based on docking-generated structures and affinities, so that it is not clear how closely it tracks experimental data. Importantly, their approach suffers from some of the well-known limitations of exact Gaussian processes. These models exhibit *O*(*N*^3^) scaling in the number of training points (or *O*(*N*^2^) in some more efficient modern implementations), and are thus completely infeasible for datasets larger than 5–10,000 sequences or so. Our variational Bayesian network-based approach does not suffer from any such limitation.

While we validated our approach using Atezolizumab as a starting point and PD-L1 as a target, there is nothing in this approach which is specific to the selected target, and thus this approach can easily be adapted to other targets and problems. The pipeline we describe can easily be modified to optimize only a single CDR or a subset of the available positions, and to incorporate other models that rank candidate sequences for other desired properties in addition to affinity. We anticipate that this pipeline and modified versions of it may therefore prove to be a useful tool for accelerated antibody discovery and development.

## Methods

### Software

Analysis and modeling was conducted using Python 3.9 with the PyTorch library version 1.8.1, the Numpy library version 1.19.5, the Scipy library version 1.5.4 and the scikit-learn library version 0.24.2. 0 was used as a random seed for model weight initialization, train-test splitting etc.

### Autoencoder model for antibody encoding

The training set used for the autoencoder is derived from the cAb-Rep database^41^, consisting of sequenced antibody repertoires from 121 human donors (https://cab-rep.c2b2.columbia.edu/tools/). For these experiments, we used the high-depth repertoire dataset. This dataset was further filtered by numbering all sequences using the ANARCI software^42^ with Chothia numbering. During this process, the ANARCI software aligns the input sequences to domain-specific hidden Markov model profiles for human antibodies using the HMMer software^64^. Any sequences with low bitscores resulting from this alignment are rejected, thereby minimizing the chance the dataset will contain proteins that are not actually antibodies. After numbering and filtering using ANARCI, 2,725,492 sequences remained. Since these sequences are from a database of antibody sequences and were filtered to select those that had a high probability of being generated by antibody MSAs, we can be quite confident they are antibodies. It should be noted that the sequences we used here are VH heavy chain only to match the sequences used in the experimental work.

The autoencoder accepts one-hot encoded sequences as input. To number the positions of each antibody, we used the Chothia numbering scheme^65^. A heavy chain may have as many as 132 amino acids in this scheme, although our mutants have fewer since the size of the complementarity-determining regions or CDRs varies between antibodies. Consequently, each one-hot encoded sequence is a matrix with 132 rows and 21 columns. While there are only 20 amino acids present, the 21st position indicates a blank, since our heavy chains do not contain the full 132 amino acids present and therefore have blanks at some Chothia-numbered positions. It is of course also possible to merely leave blank positions as all zeros, although we prefer to explicitly encode blanks as such for clarity and adopt this approach here. Each row contains a 1 at one position to indicate which amino acid (or a blank if no amino acid) is present.

Frequently in selecting and designing sequences it is important to determine which positions are most important for affinity or other desired properties. This consideration suggests the learned representation generated by the autoencoder should have the same number of rows (positions) as the input. Moreover, in order to ensure a specific row of the encoding contains information relevant to that position, the decoder should be able to reconstruct the amino acid present at each position using the information at that position (and possibly the neighboring positions).

In keeping with these constraints, the encoder portion of the model was designed to compress the input from a 132 × 21 matrix to a 132 × 3 matrix. The encoder module consists of a convolutional network with two convolutional layers with appropriate padding and a third linear layer. The first convolutional layer contains 40 kernels, each of width 21, while the second contains 20 kernels of width 11. Each convolutional layer uses gated convolutional activation, shown by Dauphin et al.^66^ to improve performance of convolutional neural networks on language modeling tasks. For this activation function, the sigmoid function is applied to the first half of the columns in the output from that layer and these are then multiplied elementwise with the second half. In other words, if the output of a layer for a given sequence is a 132 × 40 matrix, for gated convolutional activation the sigmoid function is applied to the first 20 columns, and these are then multiplied elementwise with the remaining columns to yield a final output of dimensions 132 × 20.

The final output of the linear layer in the autoencoder is a 132 × 3 matrix, which is an encoding of the original sequence. This encoding becomes the input both to a decoder module and to a prediction module. The decoder module consists of a single layer of the following form:

1 $${{{{{\rm{softmax}}}}}}(a\cdot W+b)$$

Here *a* is one row of the output of the encoder. Since the output of the encoder is a 132 × 3 matrix, *a* is then a 3 dimensional vector. *b* is a learned bias vector and *W* is a learned 3 × 21 weight matrix. Softmax is the softmax function:

2 $$\frac{{e}^{{\,z}_{i}}}{\varSigma {e}^{{\,z}_{j}}}$$

Where *z* is the 21-element vector resulting from *a*⋅*W* + *b*. The decoder layer is applied to each row of the encoder output. This design imposes a strong constraint on the autoencoder: the model is required to reconstruct the input using a decoder function with only a relatively small number of parameters shared across all positions.

The output of the encoder is also supplied to a prediction module that differentiates sequences of human B-cell receptors from junk sequences. The prediction module consists of a simple logistic regression model, where the probability that the sequence is an antibody is given by:

3 $$\frac{1}{1+{e}^{-w\cdot a}}$$

where *a* is the full output of the encoder flattened from a 132 × 3 matrix to a length 396 vector and *w* is a length 396 learned weight vector plus a learned bias term. Since both the prediction and decoder modules have few parameters (by comparison with typical deep learning models), neither can learn a complicated mapping from encoded sequence to input, thereby placing the burden on the encoder to generate as informative and relevant an encoding as possible.

To generate the junk sequences, we began with the 2,725,492 sequences selected from the cAb-Rep database and generated a mutant version of each. Our goal here is to force the model to incorporate information about the relative abundance of specific amino acids at specific positions into its encoding by requiring it to be able to distinguish true antibody sequences from closely related sequences. We could of course simply generate random sequences but this would not force the model to generate an informative encoding; random sequences are so different from antibodies that the logistic regression model which predicts whether a sequence is or is not an antibody would be able to distinguish them without any modifications to the representation generated by the encoder. It is therefore preferable to generate decoy sequences that are just similar enough they will be difficult to distinguish and yet different enough to exhibit modifications rare in true antibody sequences.

Our experiments suggested 7 mutations provided a good balance; consequently, each copy was altered to a randomly chosen amino acid at seven randomly selected positions. The end result of this process was thus a library of roughly 6 million sequences, half of which are human B-cell receptors and the other half of which are not. The autoencoder model is thus trained both to encode an input sequence and to embed information about typical features observed in true antibody sequences. The autoencoder was implemented using the PyTorch library in Python 3.6.9 and trained on the full 6 million sequence dataset until convergence. The code for this and all other steps described in this paper is available online at: https://github.com/Wang-lab-UCSD/RESP (10.5281/zenodo.7508853).

Accuracy was assessed separately for both the prediction task and the reconstruction task using a held-out test set. These metrics are used only as diagnostics because they assess the ability of the autoencoder to reconstruct its input. The true test of the autoencoder is the degree to which the encoding it generates affects predictive accuracy of a model trained with that learned representation as input. To evaluate this more critical metric, we encode the WT and mutant Atezolizumab library (construction described below) using the autoencoder described above, one-hot encoding, the ProtVec encoding scheme, the UniRep encoding scheme, the FAIR-ESM encoding scheme, the AbLang seq-coding scheme and the AntiBertY embeddings. For AbLang, we use the seq-codings option as recommended in the documentation. For the AntiBertY embeddings, we average across all the residue-specific representations in the sequence. For AntiBertY, we initially tried using all of the residue-specific embeddings without averaging across them, but found this led to poor performance. We train a Bayesian neural network (construction described below), a fully connected neural network with the same number of layers and weights as the Bayesian network and a random forest model as a baseline using these available encodings. We evaluate classification performance of each model on each encoding using 5× cross validation on the training set and a held-out test set, with Matthews correlation coefficient and AUC-ROC for identification of RH03 vs rest as metrics. A good encoding should improve or at least not damage performance relative to one-hot encoding across all three model types and especially the Bayesian network, which is the model of most importance for this study.

### Generation of the WT and mutant Atezolizumab scFv library (see also S2.2)

The WT Atezolizumab scFv^49^ was first cloned into the pYD1 yeast display vector to test its function on the yeast surface (see Supplementary Fig. 1). For the 1st Atezolizumab library, the WT plasmid was used as a template for PCR to prepare either the WT light chain with Q5 hotstart DNA polymerase (NEB) or the mutated heavy chain by error-prone PCR with Taq Polymerase (Invitrogen) as previously described^48^. The 2 PCR products were assembled into one product by overlap extension PCR and co-electroporated into EBY100 yeasts along with linearized pYD1 vector as described^67^. The library transformation resulted in 7.8 × 10^7^ transformants, based on colony counts after serial dilution onto selection plates.

### Atezolizumab scFv library screening by yeast surface display (see also S2.3)

The optimal competition time for the off-rate screens was determined as previously described^50,54^. The WT or mutant library yeasts were thawed and inoculated into selective growth media and grown at 30 °C for 22 hrs. The library/WT were induced at 20 °C in galactose induction media 42 hrs. Afterwards, WT or library was labeled with biotin-PD-L1 (Sino Biological 10084-H08H-B) 3 hrs at RT in TBS-BSA, followed by incubation with excess non-biotin-PD-L1 (Sino Biological 10084-H08H) for the determined competition period (at RT, in TBS-BSA). For FACS, yeasts were labeled with anti-V5 (Thermo Fisher R960-25, previously known as 46-0705) at 4 °C in TBS-BSA, followed by SA-PE (BD 554061) and goat anti-mouse IgG2a AF647 (Thermo Fisher A21241) on ice for 30 min in TBS-BSA. Cells were sorted for faster, moderate, and slower off-rates (see Supplementary Fig. 12). Hits were grown up to high density at 30 °C and made into frozen stocks at −80 °C.

### Preparation of the DNA libraries of mutants with faster, WT-level, and slower off-rates to PD-L1

To isolate plasmids from each binding group from the initial library screen, plasmids were harvested from yeasts by Zymoprep Yeast Plasmid Miniprep II kit (Zymo Research), eluted with ddH20, concentrated with the DCC-5 (DNA Clean and Concentrator 5) kit (Zymo Research), then subjected to the 1st round of PCR using primers SeqF/SeqR (Supplementary Table 2, designed to only amplify the heavy chain regions). The 1st PCR was performed with Q5 Hotstart DNA Polymerase (NEB) using a moderate number of PCR cycles (17, determined to be optimal by qPCR) to avoid over-amplification of the library and the PCR product concentrated with DCC-5 kit and purified by agarose gel extraction. The second PCR was carried out with various primers (Low/Medium/High Binder NGS F, NGS R) to barcode each binding group (see Supplementary Table 2) for MiSeq Nano PE250 using KAPA HiFi Hotstart Ready Mix (KK2601) for 5 cycles (determined optimal by qPCR). The PCR products were purified with Ampure XP beads (A63880) and submitted for QC using TapeStation analysis (Agilent HS D1000) before submission for MiSeq sequencing at the Institute of Genomic Medicine, UC San Diego.

### Generation and screening of the focused 21-mutant library (see also S2.4)

Twenty-one geneblock fragments (IDT) for the 21-mutant heavy chains were fused with the light chain by overlap extension PCR (Q5 hotstart), followed by co-electroporation into EBY100 yeasts with the linearized pYD1 vector as with the 1st library (>10^7^ transformants). The resulting library was screened essentially as for the 1st library, except the final sort involved a very stringent competition time (39 h at RT). The hits were harvested by yeast plasmid miniprep, transformed into GC10 competent cells, harvested by bacterial miniprep, and sequenced using standard Sanger sequencing.

### Cloning WT Atezolizumab, Mutant 4, Durvalumab, and Avelumab scFv into the pYD1 vector

The geneblocks for Durvalumab and Avelumab were purchased from IDT with yeast-optimized codons and PCR amplified using the following primers: Durvalumab with Atez LE F & Durv LE R, Avelumab with Atez LE F & Avel LE R (Supplementary Table 2). The WT/Mutant 4 genes were PCR amplified from their plasmids using Atez LE F/R primers. The PCR products were double digested using XhoI/NheI-HF (NEB) and ligated into linearized/dephosphorylated pYD1 (using the same enzymes to digest and rSAP/T4 DNA ligase (NEB) to dephosphorylate/ligate) and the ligation product transformed into GC10 competent cells (42-658, Genesee Scientific). Plasmids were isolated using a Zippy Plasmid Miniprep Kit (Zymo Research) and sequence verified before transformation into EBY100 yeasts. These constructs were used for the yeast *k*_off_ and *K*_D_ determinations.

### Determination of the WT/Mutant 4/Durvalumab/Avelumab *K*_D_ and *k*_off_ values on the surface of yeast (see also S2.5)

The *k*_off_ was determined at RT essentially as described^54^ and in section S2.3 in TBS-BSA. The resulting data was fit to a one phase decay model with GraphPad Prism 9.3.0 software using the following equation (Y is fraction of yeast bound to biotin-PD-L1, X is time, Y0 = 1 (fraction of yeast bound to biotin-PD-L1 at the 0 s competition time point), plateau is a constant based on nonspecifically bound yeast, and *K* is *K*_off_):

4 $$Y=(Y0-{{{{{\rm{Plateau}}}}}})\times \exp (-K\times X)+{{{{{\rm{Plateau}}}}}}$$

The *K*_D_ values on the surface of yeasts were determined basically as described^48^ and the data fit to the following equation:

5 $$Y=\frac{{B}_{\max }X}{{K}_{d}+X}$$

(Bmax is maximum MFI value, X the concentration of PD-L1). Due to the very slow off-rate of Mutant 4, it was necessary to incubate yeasts with PD-L1 for 6 days at RT.

### Cloning and purification of WT/Mutant 4 Atezolizumab scFv (also see S2.6)

The scFv sequences were PCR amplified from the pYD1 vector and cloned into the pET27b(+) (69863-3, MilliporeSigma) vector for bacterial expression. The vectors were transformed into Rosetta(DE3) cells (70954-3, MilliporeSigma) and the scFv-His_6_ fusions purified essentially as described^68^. The scFv stocks were frozen in PBS + 10% glycerol at −80 °C. SDS-PAGE was used to assess purity (Supplementary Fig. 5) and protein concentration determined by BCA assay (Pierce #23227).

### Determination of the *K*_D_ values by bio-layer interferometry (BLI)

BLI was performed at the Biophysics and Biochemistry Core at The Scripps Research institute on an Octet Red96 at 25 °C. Binding reactions were performed in 1× kinetic buffer (Sartorius, 18-1105) consisting of 20 mM phosphate buffer, pH 7.6, 2 mM KCl, 150 mM NaCl, and 0.02% Tween 20, 0.1% BSA, .05% sodium azide. Biotinylated PD-L1 (Sino Biological 10084-H08H-B) was immobilized on SA Biosensors (Sartorius, 18-5019) by dipping the sensor into 100 nM b-PD-L1 until the signal was saturated. A blank loading channel was used as a negative control. Kinetic experiments were performed with both scFv WT and scFv Mutant 4. Experiments were performed using a gradient of concentrations, with the scFv WT ranging from 475 nM to 0.6 nM, and scFv mutant ranging from 160 nM to 0.2 nM. Binding kinetics were assessed via Octet Data Analysis HT Software Version 12 using a 2:2 binding model.

### Mass photometry

Mass photometry was performed on a Refeyn TwoMP at the Scripps Research Institute, at room temperature. The PD-L1 used was biotin-PD-L1 (SinoBiological 10084-H08H-B) and purified scFv of the WT or Mutant 4. Each characterization was performed in 1X PBS (phosphate buffer from Cytiva BR100672). For each experiment, the scFv or PD-L1 (or both) were diluted with PBS to the final concentration. Each experiment resulted in a 60 s movie, and mass analysis performed using Refeyn DiscoverMP analysis software v2.3.0. Mass calibration was performed using Urease (Sigma, U7752) and Thyroglobulin (Millipore, 609310).

### Sequence processing

Raw paired end reads from the Atezolizumab dataset were checked for quality (for details of the filtering criteria, see the Supplementary Information section S2.1). After the sequences had been processed, they were split into an 80% training set and a 20% test set. All training and cross validation was performed on the 80% training set only.

In many cases, sequences occurred in more than one category but with a different frequency in each. Clearly, our level of confidence in category assignment is reduced when the frequency of the sequence in the assigned category is not much greater than its frequency in others. To encode our level of confidence, we weight each sequence with the frequency in the assigned category plus one divided by the total number of occurrences plus three. This is the posterior probability using a multinomial likelihood and a uniform Dirichlet prior (a Dirichlet distribution with α = [1,1,1]).

The ordinal regression model used here employs binary cross-entropy loss:

6 $$\mathop{\sum }\limits_{i}^{N}\mathop{\sum }\limits_{j}^{M}-\left({y}_{i,j}\log \left({p}_{i,j}\right)+\left(1-{y}_{i,j}\right)\log \left(1-{p}_{i,j}\right)\right)$$

where *p* for datapoint *i* is the model-assigned probability that sequence belongs to a more stringent/tighter-binding category than category *j*, out of *M* possible categories and for *N* data points, and *y*(*i*,*j*) is either 0 or 1 and indicates whether the sequence does or does not belong to a tighter-binding category. (For details of how *p* is calculated, see the next section). This loss function for sequence *i* is multiplied by the weight for sequence *i* so that the model is more weakly penalized for misclassifying sequences where our confidence in the category assignment is low.

### Construction of the ordinal regression model

**Fig. 9:**
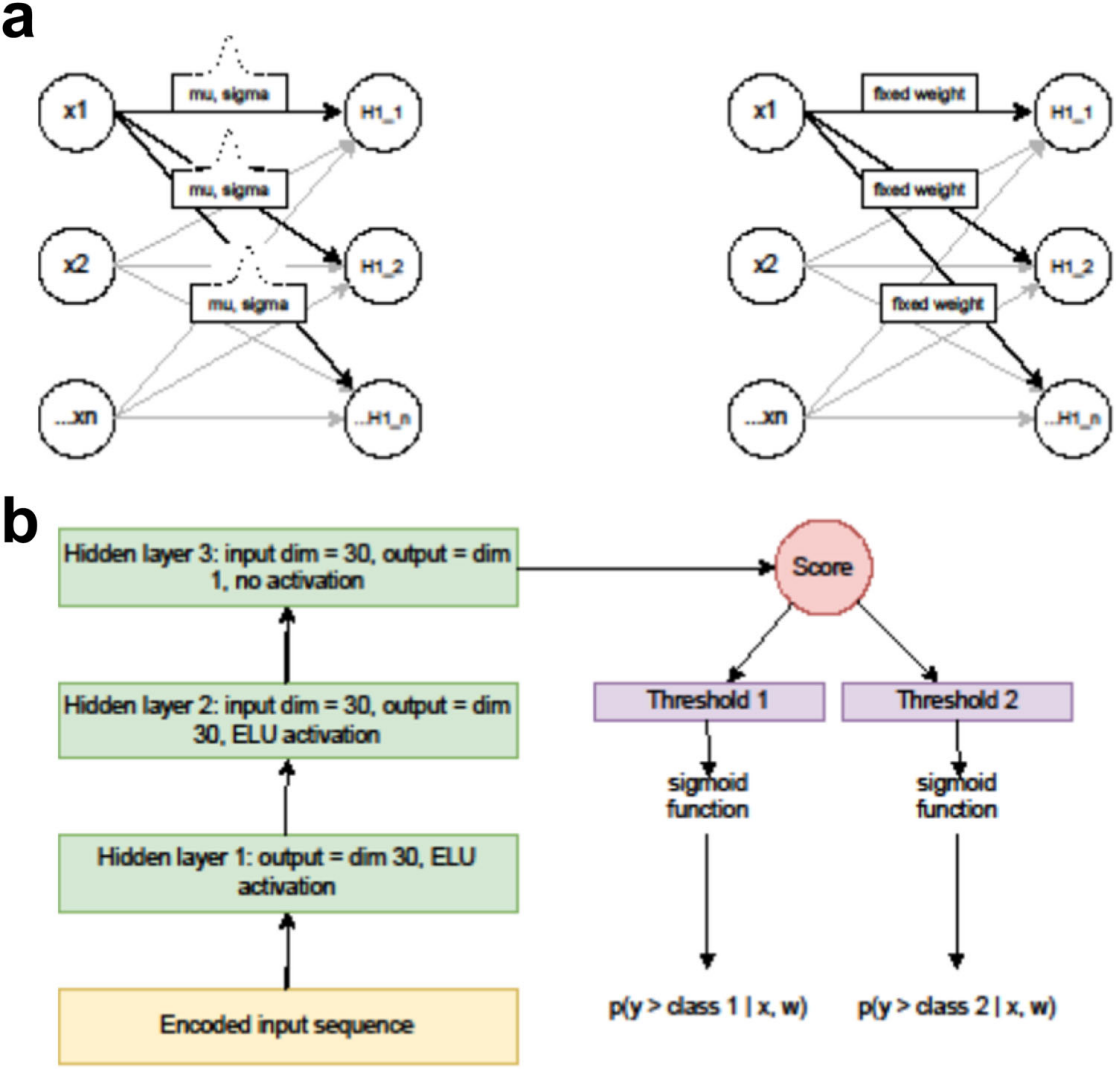
The variational Bayesian neural network architecture. **a** A comparison of a variational Bayesian neural network with a fully connected architecture. Both networks map a vector of input values x1, x2,…xn to a hidden layer vector H1_1, H1_2,…H1_n by a matrix multiplication followed by a nonlinear activation. In the fully connected network, however, each element of the hidden layer weight matrix is a learned value and once the network is trained it is a fixed value. In the Bayesian network, by contrast, each element of the hidden layer weight matrix is a Gaussian distribution specified by a learned mean and a learned standard deviation. To generate predictions, we can sample from the weight distributions, which provides an estimate of the uncertainty on our predictions. **b** The structure of the ordinal regression scoring model used in the pipeline.

The Atezolizumab mutant library data was analyzed using Bayesian neural network-based ordinal regression. Each mutant Atezolizumab sequence is first encoded by the autoencoder, yielding a 132 × 3 matrix which becomes the input to the Atezolizumab model. The model architecture is illustrated in Fig. 9. At a high level it is similar to the Bayes by Backprop architecture described by Blundell et al.^51^, except our model has been adapted to perform ordinal regression as described below. We now walk through the architecture in more detail.

In a traditional neural network, each parameter is a learned parameter that is fixed once the model is trained. In the variational network, by contrast, each parameter has an associated Gaussian distribution described by a mean and a standard deviation whose optimal values we learn during training. To generate predictions, rather than using the fixed learned parameters as in a traditional network, we sample from the weight distributions *N* times to generate *N* predicted values. The variance in these predictions provides a measure of our uncertainty around the final prediction. This approach requires a more complicated training procedure than that associated with traditional neural nets which we now discuss.

Briefly (for a full derivation see ref. ^51^), we seek parameters *θ* for a distribution on the weights *w*, that will minimize the evidence lower bound or ELBO given by:

7 $${{{{{{\rm{argmin}}}}}}}_{\theta }{KL}[q(w{{{{{\rm{|}}}}}}\theta ){{{{{\rm{|| }}}}}}p(w)]-{E}_{q(w{{{{{\rm{|}}}}}}\theta )}[\log (p(D{{{{{\rm{|}}}}}}w))]$$

where $q({w|}\theta )$ is a distribution over the weights that approximates the true Bayesian posterior, *p*(*w*) is the prior and $p({D|w})$ is the likelihood of the data. The parameters *θ* are the parameters of the normal distributions for all of the weights in each hidden layer as illustrated in Fig. 9. The second term is the negative log likelihood, while the first term is a regularization term that measures the divergence between the weight distribution learned by the model and the prior. We chose a Cauchy prior with unit scale and location zero, which adds some additional flexibility since some of the weights are now expected to be outliers.

We approximate the cost via a Monte Carlo sampling procedure. Using the current set of parameters *θ* (the means and standard deviations of the weight distributions), for each minibatch we draw *n* sample sets of weights to yield the following approximate cost function:

8 $${{{{{\rm{Loss}}}}}}\approx (\frac{1}{{nM}})\mathop{\sum}\limits_{i}^{n}{{\log}}\left(q\left({w}_{i},| , \theta \right)\right)-{{\log}}\left(p\left({w}_{i}\right)\right)-\log (p({D|}{w}_{i}))$$

where *M* is the number of minibatches and the other terms are as above. In other words, for each minibatch of training data, we draw *n* sets of sample weights and then average the approximate cost function across these and across the minibatches in the training set. Backpropagation for this cost function is made tractable by using the reparameterization trick of Kingma and Welling^69^. We sample from a standard normal distribution with mean zero, standard deviation 1, then add the mean and multiply by the standard deviation of the distribution for weight *j*. In order to ensure that the standard deviation is always positive, instead of using the standard deviation itself as a parameter, we parameterize each distribution with a parameter *ρ* that is converted to the standard deviation using the softplus function:

9 $$\sigma=\log (1+{e}^{\rho })$$

By using this reparameterization trick, the gradient of the approximate cost function with respect to the mean and *ρ* of the distribution for each weight and bias term in each hidden layer is easily calculated. The advantages of this model structure are two folds. First, it imposes strong regularization on the model parameters that as demonstrated by Blundell et al.^51^ can provide improved performance for some tasks. Second, it enables us to estimate the uncertainty in our predictions and thereby ascribe greater weight to the most confident predictions when selecting sequences for testing.

All these features of our model are shared in common with most Bayesian neural network architectures. We however use our model instead to perform ordinal regression. As illustrated in Fig. 9, the last hidden layer of the Bayesian network outputs a single latent score value. This score is added to *M − 1* learned threshold values for the case where there are *M* categories. The sigmoid function is then applied to each of the *M − 1* outputs to generate an output vector of *M − 1* probability values. Each element *i* of this output vector is the model-assigned probability that the input sequence belongs to a binding category more stringent than *i*. Since there are three binding categories (RH01, RH02 and RH03 or weak, moderate and strong), the output vector is 2-dimensional; the first element indicates the probability that the sequence belongs to either RH02 or RH03, while the second element indicates the probability the sequence belongs to RH03. The model is trained by minimizing the binary cross-entropy loss described above.

This arrangement treats the categories as ranked: sequences with a higher latent score are thereby assigned to higher categories^70^. Note that it does not predict the actual off rate or binding affinity. Rather, the score reflects our confidence the sequence will be a strong binder relative to others in the training set. This approach has been used previously by Parkinson et al. to rank sequences on a protein engineering task and select them for experimental evaluation^40^.

### Sequence scoring and selection

We modify the classic simulated annealing algorithm and equip it with our trained models to perform in silico directed evolution as illustrated in Fig. 3. First, we compute the frequency of each amino acid at each position across the entire dataset, add 1 to all values and divide by the total number of sequences in the dataset plus 20 to retrieve a marginal probability for each amino acid at each position. By adding 1 to all frequencies, we ensure there is a small but nonzero marginal probability for amino acids not observed in the dataset.

Next, we select the 500 highest-scoring sequences in the Atezolizumab dataset and find the top 10 most frequently mutated positions in these sequences. The selection of 10 sites here is arbitrary; we could use more or fewer if desired. On each iteration, we select with equal probability any of these top ten sites. The selected site is randomly reassigned to a new amino acid; the probability for the selection of any given new amino acid is determined by the marginal probabilities calculated as described above. Assume, for example, that 80% of all sequences observed in the dataset carry an arginine at position 100, another 10% carry lysine and so on. If position 100 is selected on a given iteration, this position will be mutated with an 80% chance of being converted to an arginine, a 10% chance of being converted to a lysine and so forth.

The current sequence and the mutated proposed sequence are both encoded using the autoencoder and the trained ordinal regression model is used to assign a score to each. The proposed sequence is accepted with a probability given by:

10 $$p({{{{{\rm{acceptance}}}}}})={e}^{\frac{-({S}_{{{{{{\rm{best}}}}}}}-{S}_{{{{{{\rm{proposed}}}}}}})}{T}}$$

Where *T* is the temperature and *S*_best_ and *S*_proposed_ are the best score to date and the score of the proposed sequence respectively.

For a further illustration of this algorithm, refer to Supplementary Fig. 6. This procedure is a simple approach for exploring the sequence space while ensuring we do not venture too far from the training set. To ensure reproducibility and avoid stochastic fluctuations, for this stage of modeling the Bayesian neural network generated predictions using the mean of the distribution for each weight as the weight value.

### Statistics and reproducibility

No statistical method was used to predetermine sample size. When processing raw sequence data, unreliable sequence reads (reads containing one or more bases with a phred quality score <10 or where the paired end reads did not match in the overlap region) were discarded before any further analysis or processing was conducted. These steps were taken to ensure that only reliable reads were used for analysis. No data was otherwise excluded from any subsequent analysis or model training. The test set for evaluating model performance was constructed by randomly selecting 20% of the assembled sequences and assigning these to test. The random partition was generated using the Mersenne Twister random number generation algorithm as implemented in Python’s numpy library version 1.19.5 with a seed value of 0. When model performance was assessed using cross-validations, the cross-validation splits were generated by randomly partitioning the dataset into 5 splits of equal size using the KFold function in Python’s scikit-learn library version 0.24.2.

The final evaluation of model performance was conducted “blind” by generating predictions for sequences not present in our data and experimentally evaluating these predictions as described above.

### Reporting summary

Further information on research design is available in the Nature Portfolio Reporting Summary linked to this article.

## Supplementary information

Supplementary Information

Reporting Summary

## Data Availability

The raw sequence read data generated for this study has been uploaded to the Sequence Read Archive (SRA) database under accession code PRJNA813220. The antibody sequence data used to train the autoencoder used in this study are available in the cAbRep database [https://cab-rep.c2b2.columbia.edu/]. The construction of the cAbRep database is described in Guo et al.^41^. Source data are provided with this paper.

## Source data

Source Data

## Code availability

The code used in this study is available online at https://github.com/Wang-lab-UCSD/RESP (10.5281/zenodo.7508853), together with instructions on how to reproduce all key computational experiments.
