# Supplementary material for: The RESP AI model accelerates the identification of tight-binding antibodies
Source: Nat Commun. 2023 Jan 28;14:454. doi: 10.1038/s41467-023-36028-8 (PMC9884274; doi:10.1038/s41467-023-36028-8)
Supplement: Supplementary file 1 — Supplementary Information [file 41467_2023_36028_MOESM1_ESM.pdf]

RESP: An AI model to accelerate identification of tight-binding antibodies

SUPPLEMENTARY INFORMATION

Supplementary Figure 1: WT Atezolizumab scFv binds human PD-L1 on the yeast surface. The y-axis of each panel represents expression of the scFv (V5 epitope/AF647), while the x-axis the binding intensity to human PD-L1/PE intensity. Numbers of double positive cells out of the total analyzed are given below each panel.

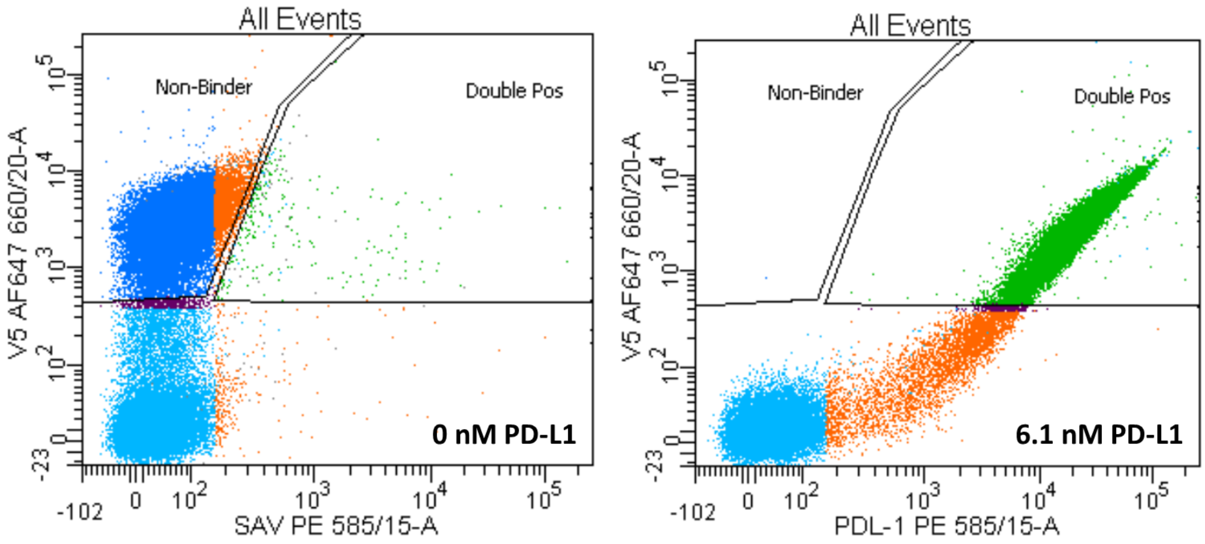

236/50,000 are double positive cells

26,504/50,000 are double positive cells

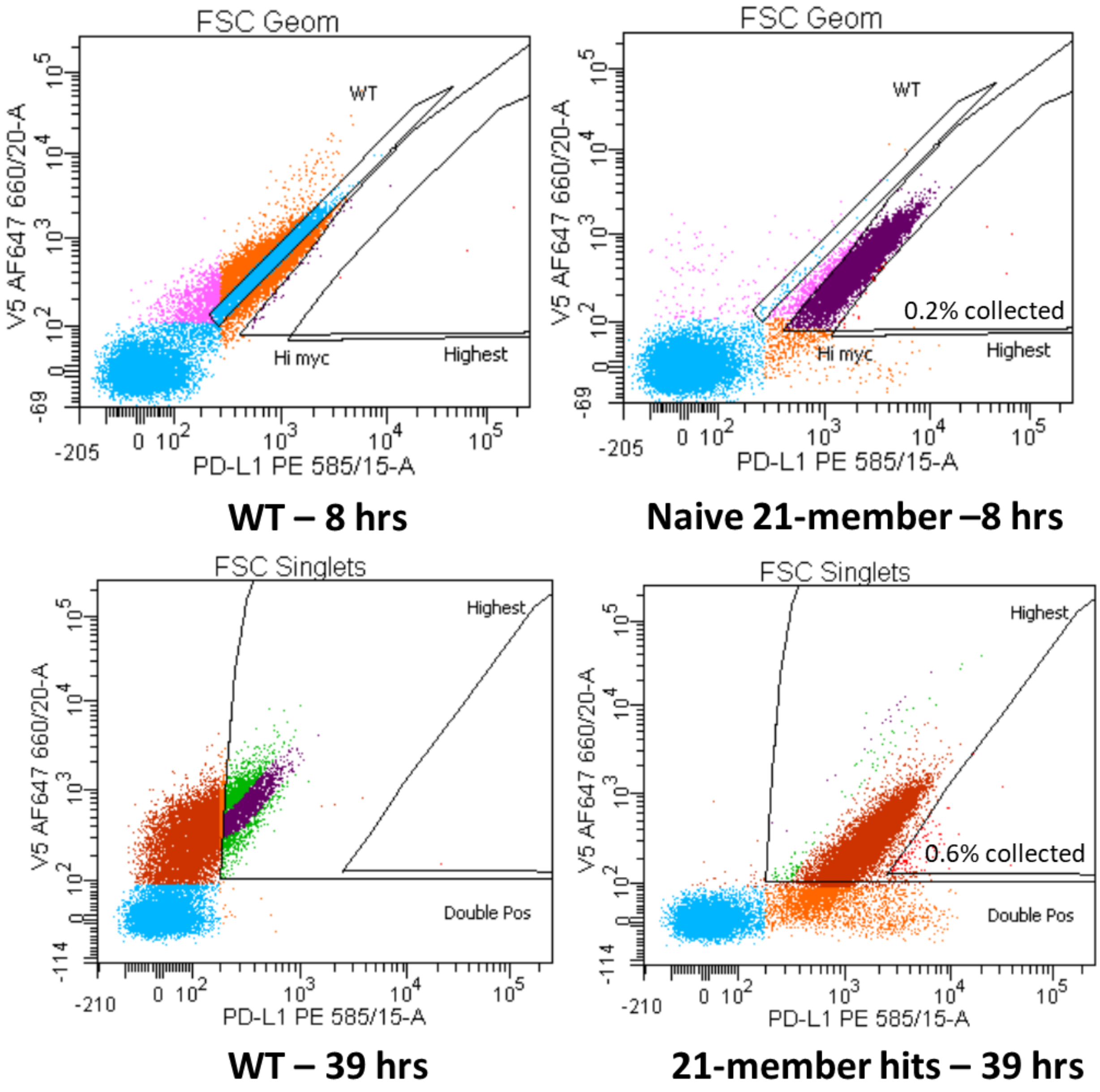

Supplementary Figure 2. First screen of the 21-member library after 8 hours off-rate competition (relative to WT, top plots) and final screen of the library after 39 hours competition (bottom plots). The right triangle represents the sort gate with associated % of cells collected per sort. The y-axis of each panel represents expression of the scFv (V5 epitope/AF647), while the x-axis the binding intensity to human PD-L1/PE intensity.

A

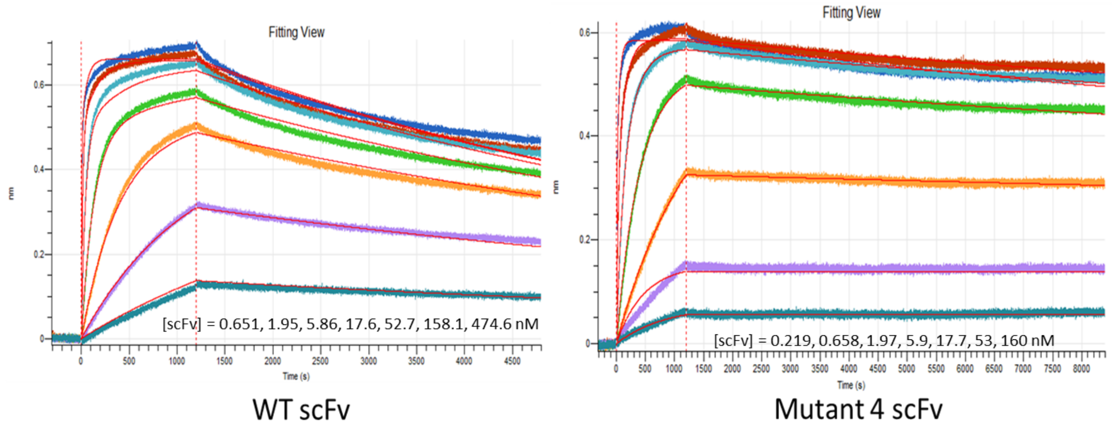

B

scFv	K_{ON}	K_{OFF}	K_D (nM)
WTa	4.31 X 10^5 $M^{-1}s^{-1}$	9.44 X 10^{-5} s^{-1}	0.219
WTb	3.87 X 10^4 $M^{-1}s^{-1}$	2.30 X 10^{-4} s^{-1}	5.93
MTa	ND	ND	ND
MTb	ND	ND	ND

a = Primary (major) signal values
b = Minor/weaker signal values
MT = mutant, ND = cannot determine

Supplementary Figure 3. BLI binding analysis of soluble WT and Mutant 4 scFv against immobilized biotin PD-L1. a) Binding curve (association and dissociation) profiles for different scFv concentrations. WT was titrated over a range from 0.65 to 475 nM while Mutant 4 over a range of 0.219 to 160 nM (different colors indicate different concentrations of scFv, with higher concentrations leading to higher y-axis values). b) On/Off-rates and K_D for WT and Mutant 4 scFv. For each scFv, two different K_D values were obtained from 2 binding events against PD-L1. The Mutant 4 scFv values were not obtainable due to the very slow off-rate.

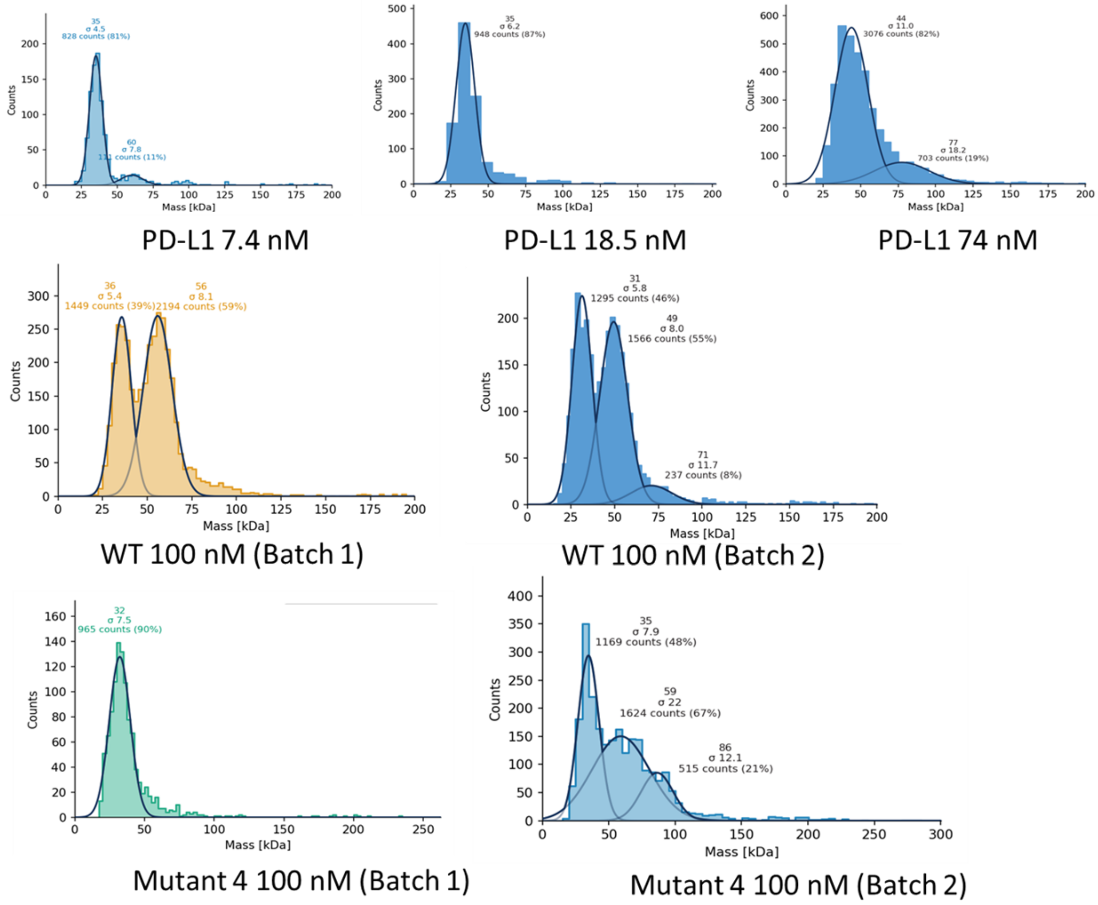

Supplementary Figure 4. Mass photometry analysis of the monomer/dimer/multimer state of PD-L1 and WT/Mutant 4 scFv. Each panel image has approximate MW (x-axis) and counts (y-axis). PD-L1 MW (theoretical) = 35-38 kDa (range due to glycosylation) and scFv = 31.4 kDa. Batches 1 and 2 are scFv from 2 separate purifications. The sigma symbol represents the standard deviation of the MW estimate as calculated from the software, the top number above each peak is apparent MW.

Supplementary Figure 5: SDS-PAGE gels (4-20%) of purified scFv-His$_6$ fusions. M4 is mutant 4. The two gel images represent proteins from 2 separate purifications (2 separate SDS-PAGE gels from 2 separate purifications). Source data are provided as a source data file containing raw gel images.

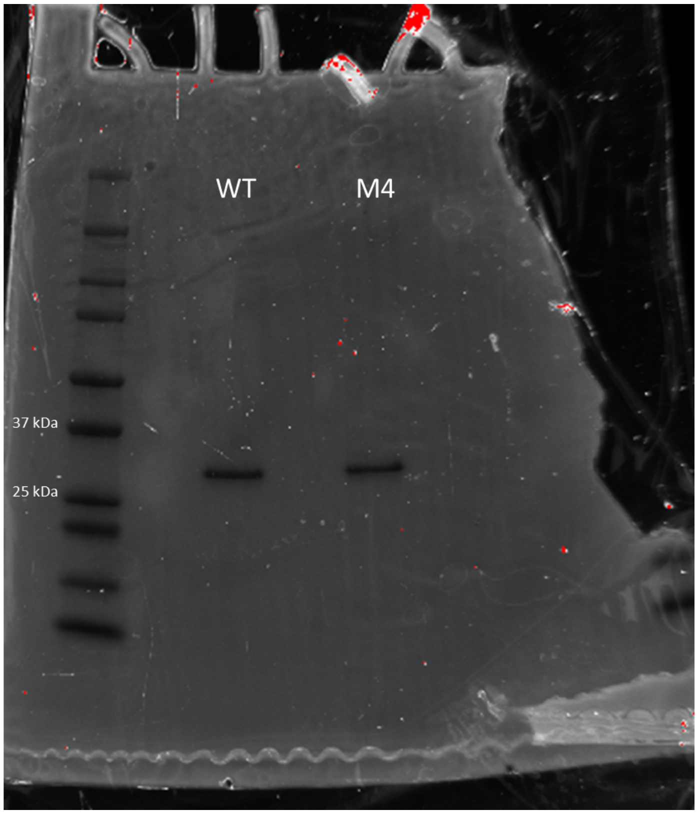

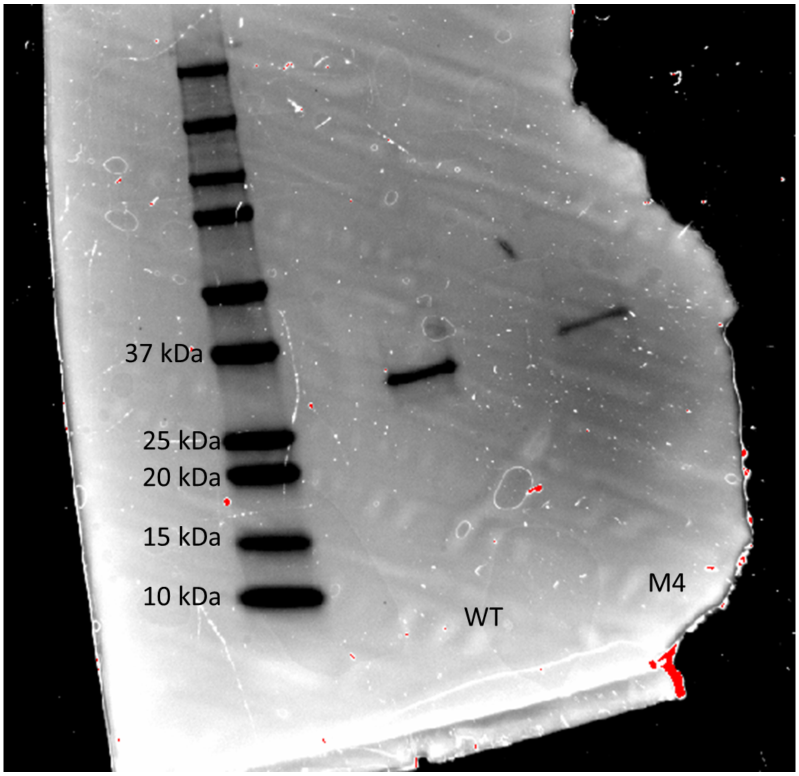
37 kDa
25 kDa
20 kDa
15 kDa
10 kDa
WT
M4

Supplementary Figure 6: Modified simulated annealing

**Algorithm**
**Input:** Frequency of each amino acid at each position in the training set plus one, divided by total number of sequences plus twenty, yielding a matrix *x* where each row *i* is the probability of each amino acid at that position; wild-type sequence; trained autoencoder and trained atezolizumab model; 10 selected positions for *in silico* mutagenesis.<br>**Initialize:** Temperature to 25.0; current sequence to wild-type atezolizumab; current score as score of wild-type sequence from the model; list $L_{accepted}$ of accepted sequences (empty).<br><br>While temperature > 0.01<br>Out of the 10 positions, randomly select position *i* for mutation<br>Select a new amino acid by sampling from $Cat(x_i)$<br>Encode the modified sequence using the autoencoder<br>Score the modified sequence using the model (in MAP mode to avoid stochasticity)<br>If sample from U(0,1) < $e^{-(current\ score\ -\ candidate\ score)/temperature}$:<br>$L_{accepted}$.append(candidate sequence)<br>Current score = candidate score<br>Current sequence = candidate sequence<br>Reduce temperature by fixed amount

Supplementary Figure 7: Binding curves between PD-L1 and yeast surface scFv. MFI is mean fluorescent intensity, M4 means Mutant 4. Each experiment is a separate/independent assay performed on a separate day. Source data are provided as a source data file.

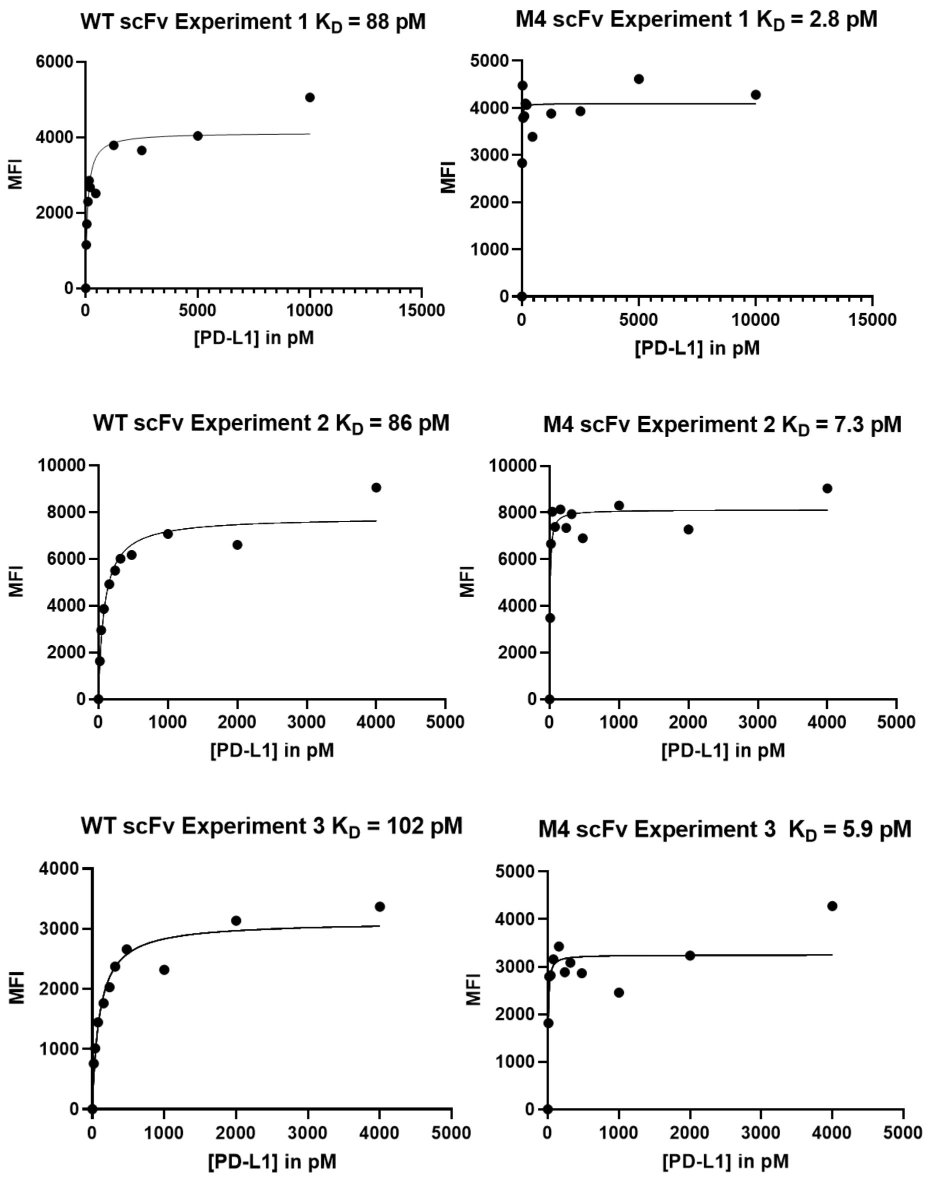

Supplementary Figure 8: Side-by-side comparison of amino acids close to R98C in the wild type (PDB structure 5XXY) and the IgFold predicted structure for the top scoring mutant. The R98C mutation is highlighted in orange and labeled, and the antigen in the PDB complex is in gray. Amino acids within 5 angstroms of R98C that have side chains in close proximity are highlighted. CDR H3 is highlighted in green for clarity.

A. The wild type

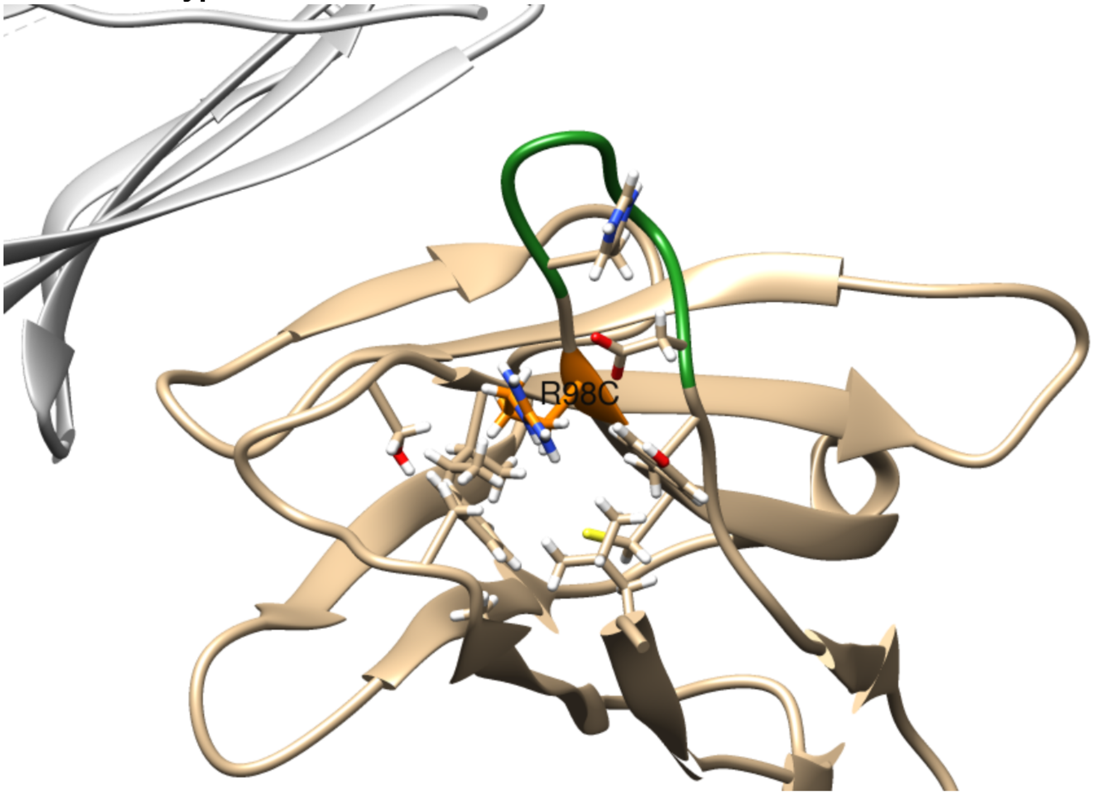

B. The top scoring mutant, A[40]V,T[58]A,I[70]A,N[77]D,A[79]I,A[97]V,R[98]C

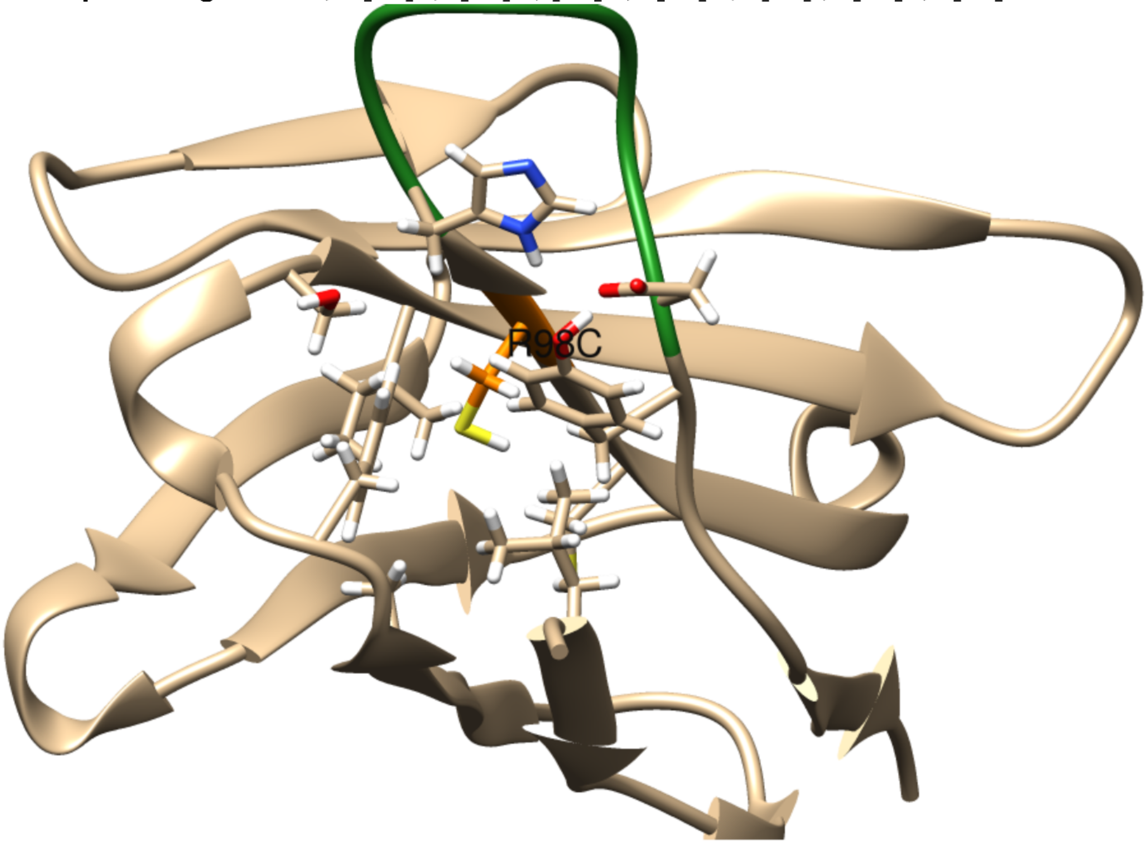

Supplementary Figure 9: Overlay of PDB structure 5XXY with the IgFold predicted structure for the top-scoring mutant. The antigen is highlighted in gray, the top-scoring mutant is in blue, the wild-type heavy chain is in cream. Note the large shift in conformation in CDR H3 and the more subtle shift in conformation in the other region that makes contact with the antigen.

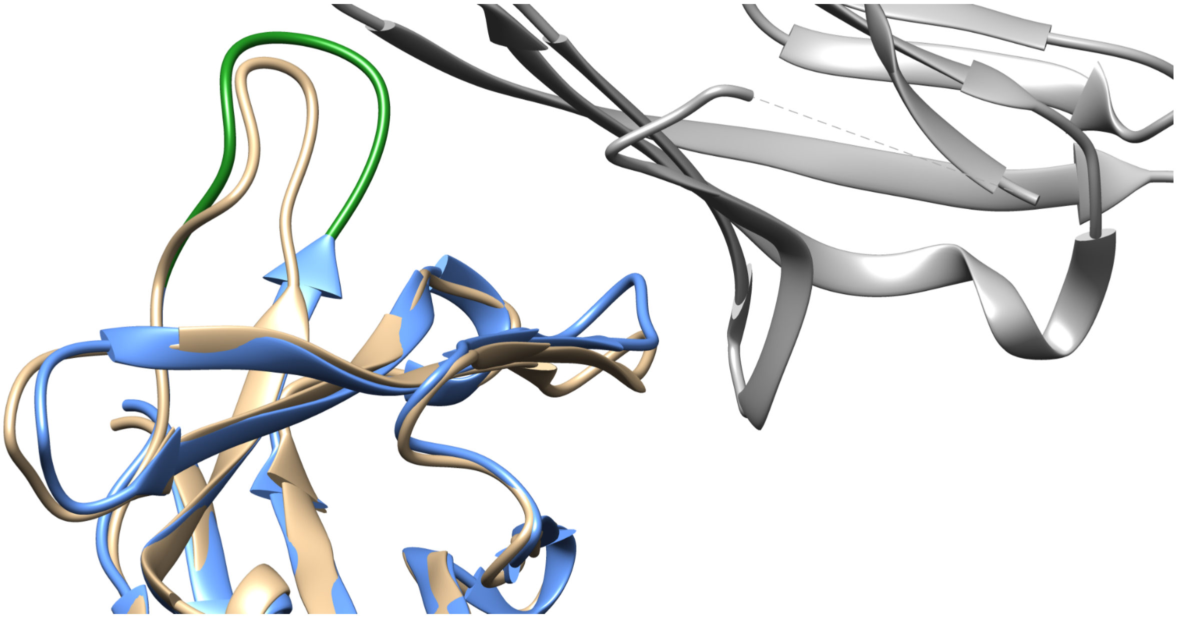

Supplementary Figure 10: Model assigned scores for sequences from Mason et al. a) The distribution of scores assigned by the model for binding and nonbinding sequences from the training set. 0 is the cutoff at which a sequence is predicted to be a binder. b) The distribution of scores for sequences selected by Mason et al as likely binders. Experimentally Mason et al. demonstrated that all of these sequences bind with K_D < 20 nM, and all of them are indeed predicted to be binders by our model. c) The model's uncertainty regarding its assigned score for predictions on the test set. Incorrect predictions have higher associated uncertainty than correct predictions, indicating that as anticipated, model assigned uncertainty can assist in determining whether a prediction should be considered reliable. There are 3,158 unique sequences in this test set. Significance was assessed using a two-sided Mann-Whitney U test; the resulting p-value was 1e-23.

The following conventions apply for each boxplot. The upper and lower bounds of the box are the 25th and 75th percentile of the data, and the whiskers are drawn at 1.5x the interquartile range (the distance from the 25th percentile to the 75th percentile). The center is drawn at the median of the data, and the "notch" represents the 95% confidence interval on the median (as determined by nonparametric bootstrap). The diamonds represent "flier" points which lie outside 1.5x the interquartile range. Four asterisks indicates the p-value is < 0.0001 . Source data is provided as a source data file.

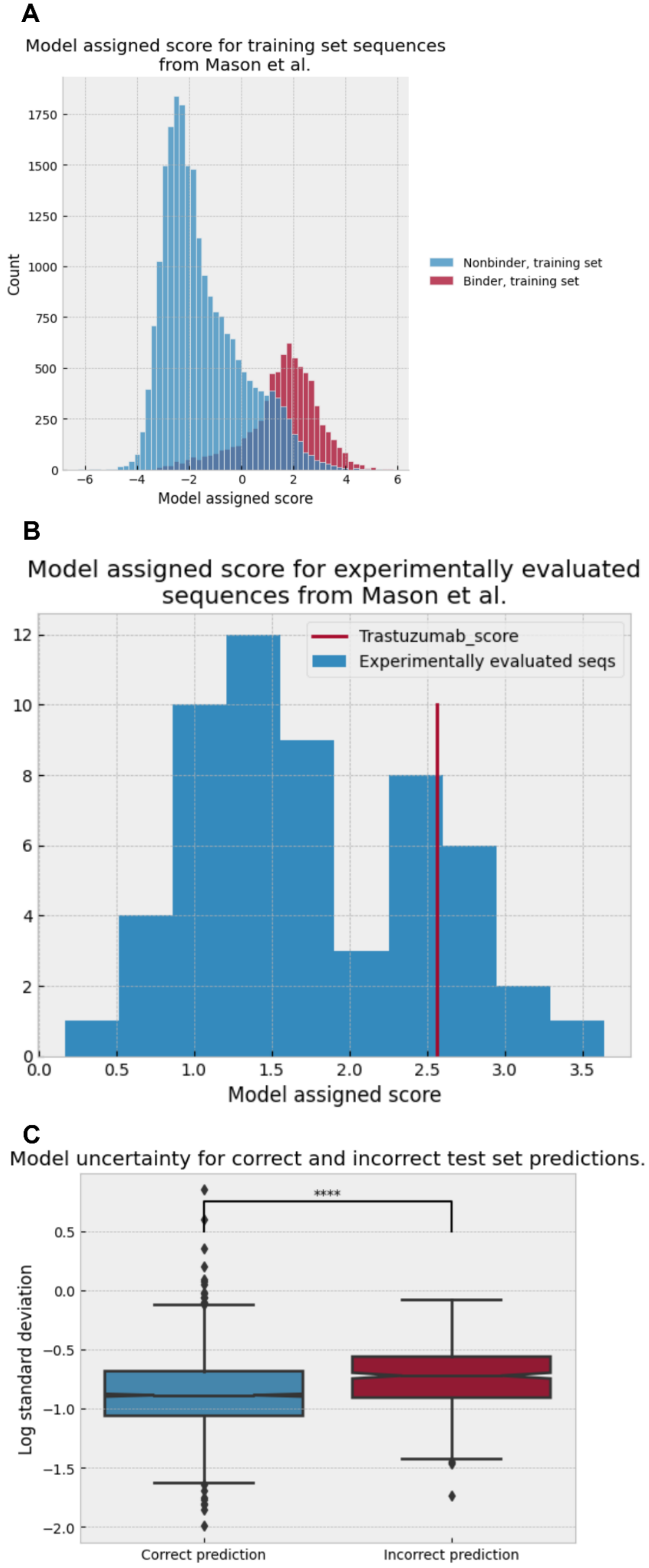
A
Model assigned score for training set sequences from Mason et al.
Count
Model assigned score
Nonbinder, training set
Binder, training set
B
Model assigned score for experimentally evaluated sequences from Mason et al.
Trastuzumab_score
Experimentally evaluated seqs
Model assigned score
C
Model uncertainty for correct and incorrect test set predictions.
****
Log standard deviation
Correct prediction
Incorrect prediction

Supplementary Figure 11: Clustering of simulated annealing results. The dendrogram for clustering of sequences harvested from the simulated annealing procedure after sequences with scores associated with wide confidence intervals had been removed. The dendrogram suggests the presence of at least two main regions of sequence space identified by the modified simulated annealing algorithm. Source data is provided as a source data file.

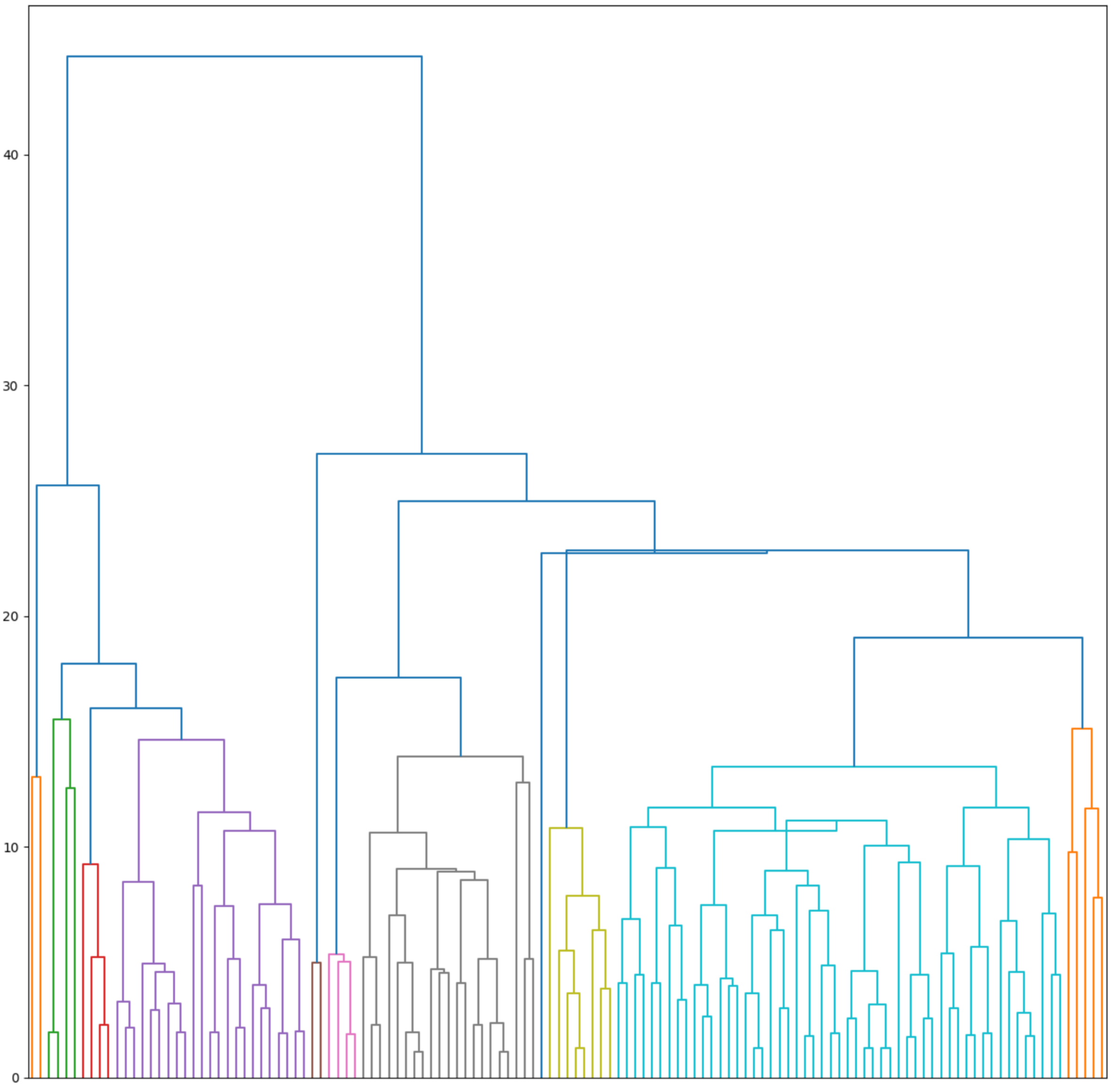

Supplementary Figure 12: Example of FACS Gating Strategy

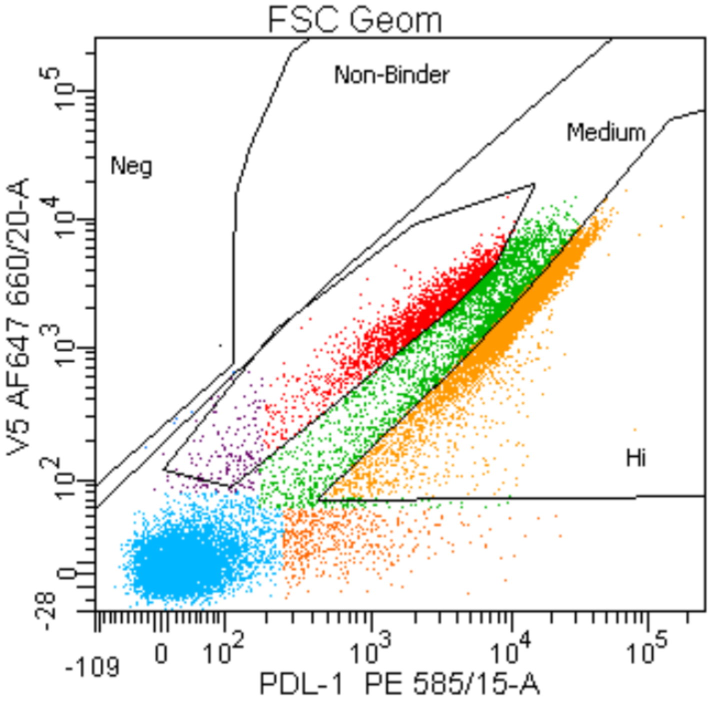

10^6 cells (orange cells, “hi” gate) collected out of 6 x 10^6 sorted yeasts. Y-axis is V5 epitope expression and X-axis is PD-L1 binding. V5 tag monoclonal mAb detected with secondary-AF647 polyclonal IgG. Biotin-PDL1 detected with SA-PE reagent.

Supplementary Tables

Supplementary Table 1: Read counts by sort category

Category	Raw reads	Accepted reads	Unique sequences	Final accepted sequences
RH01 (weak binders - faster k_{off})	290156	184094	40197	34439
RH02 (moderate binders - WT k_{off})	257076	178994	31690	26122
RH03 (strong binders - slower k_{off})	288421	187127	20666	15070

Supplementary Table 2: WT Atezolizumab, Mutant 4, Durvalumab, and Avelumab scFv geneblocks, cloning primers, primers used to generate the mutant library and DNA library for next-generation sequencing.

Geneblock/Primer	Sequence
WT Atez. Geneblock	GACATACAAATGACTCAAAGCCCGAGTTCCCTATCTGCGTCTGTTGGGGA CCGTGTTACGATTACGTGTAGAGCATCACAAGACGTTTCAACTGCGGTAG CATGGTACCAACAAAAGCCCGGTAAGGCACCCAAGCTACTGATCTATAGC GCATCTTTTCTTTACAGTGGTGTTCCGAGCAGGTTCAGTGGATCAGGGTC AGGCACTGATTTCACCTTGACCATCTCCAGTCTGCAACCGGAAGATTTCG CAACTTATTATTGCCAACAATATTTGTATCACCCAGCTACGTTCGGTCAAG GTACAAAGGTAGAAATCAAACGTGGGGGAGGTGGATCTGGGGGCGGTG GGTCCGGAGGAGGAGGATCAGGCGGTGGCGGATCTGAAGTCCAGCTTG TAGAGAGTGGGGGCGGATTGGTCCAGCCCGGTGGGTCATTGCGTTTATC ATGTGCAGCCAGTGGGTTCACCTTTAGCGACAGTTGGATCCATTGGGTAA GACAGGCTCCAGGCAAGGGCTTAGAATGGGTAGCATGGATAAGCCCCTA CGGGGGATCTACTTATTACGCTGATAGTGTAAAGGGACGTTTCACAATATC AGCTGATACGTCTAAGAACACCGCTTACCTACAAATGAACTCCCTTAGAG CCGAAGATACAGCCGTTTACTACTGTGCTCGTCGTCATTGGCCCGGAGG GTTTGATTATTGGGGGCAAGGCACATTGGTAACAGTGAGTAGT
Mutant 4 Gene	gac ata caa atg act caa agc ccg agt tcc cta tct gcg tct gtt ggg gac cgt gtt acg att acg tgt aga gca tca caa gac gtt tca act gcg gta gca tgg tac caa caa aag ccc ggt aag gca ccc aag cta ctg atc tat agc gca tct ttt ctt tac agt ggt gtt ccg agc agg ttc agt gga tca ggg tca ggc act gat ttc acc ttg acc atc tcc agt ctg caa ccg gaa gat ttc gca act tat tat tgc caa caa tat ttg tat cac cca gct acg ttc ggt caa ggt aca aag gta gaa atc aaa cgt ggg gga ggt gga tct ggg ggc ggt ggg tcc gga gga gga gga tca ggc ggt ggc gga tct gaa gtc cag ctt gta gag agt ggg ggc gga ttg gtc cag ccc ggt ggg tca ttg cgt tta tca tgt gca gcc agt ggg ttc acc ttt agc gac agt tgg atc cat tgg gta aga cag gct cca ggc aag ggc tta gaa tgg gta gca tgg ata agc ccc tac ggg gga tct act tat tac gct gat agt gta aag gga cgt ttc aca gca tca gct gat acg tct aag aac acc act tac cta caa atg aac tcc ctt aga gcc gaa gat aca gcc gtt tac tac tgt gtt cgt cgt cat tgg ccc gga ggg ttt gat tat tgg ggg caa ggc aca ttg gta aca gtg agt agt
AtezF	ACAATTCGTCTCGGTACCAGACATACAAATGACTCAAAGCCCGAGTTCC
AtezR	ATAATTCGTCTCCTCGAGACTACTCACTGTTACCAATGTGCCTTGCC
IF1F	GACATACAAATGACTCAAAGCCC
IF1R	GCTAAAGGTGAACCCAC
IF2F	GCAGCCAGTGGGTTCACCTTTAG
IF2R	ACTACTCACTGTTACCAATGTGCCTTG
OF	CGGGATCTGTACGACGATGACGATAAGGTACCAGGATCCAGTGACATACA AATGACTCAAAGCCCGAGTTCCC
OR	GAGAGGGTTAGGGATAGGCTTACCTTCGAAGGGCCCTCTAGAACTACTCA

	CTGTTACCAATGTGCCTTGCCC
SeqF	TCGTCGGCAGCGTCAGATGTGTATAAGAGACAGgaagtccagcttgtagag
SeqR	GTCTCGTGGGCTCGGAGATGTGTATAAGAGACAGagaactactcactgttaccaatg
Low Binder NGS F	CAAGCAGAAGACGGCATACGAGATACCCAGCAGTCTCGTGGGCTCGGAG<br>ATGT
Medium Binder NGS F	CAAGCAGAAGACGGCATACGAGATAACCCCTCGTCTCGTGGGCTCGGAG<br>ATGT
High Binder NGS F	CAAGCAGAAGACGGCATACGAGATCCCAACCTGTCTCGTGGGCTCGGAG<br>ATGT
NGS R	AATGATACGGCGACCACCGAGATCTACACTAGATCGCTCGTCGGCAGCG<br>TCAGATGTG
Atez LE F	AGAGCTAGCATGACTGGTGGACAG
Atez LE R	CTTGCTCGAGACTACTCACTGTTACCAATGTGCCTTG
Durv LE R	TCTTGCTCGAGACTTGAGACAGTCACAAGAGTCC
Avel LE R	TCTTGCTCGAGAGAAGATACTGTAACTAGAGTTCCCTG
scFv F	ataccccatggccgacatacaaatgactcaaagcccgag
scFv R	atacctcgagactactcactgttaccaatgtgcc
Durvalumab scFv geneblock	GCTAGCATGACTGGTGGACAGCAAATGGGTCGGGATCTGTACGACGATG<br>ACGATAAGGTACCAGGATCCAGTGAAATTGTCCTGACACAGTCACCAGGT<br>ACATTATCACTATCTCCGGGAGAAAGGGCGACACTGTCTTGTAGGGCCTC<br>ACAACGTGTCAGTTCCAGTTATTTAGCCTGGTATCAGCAGAAGCCTGGGC<br>AGGCACCTAGGCTATTGATTTATGATGCTAGTTCAAGGGCGACGGGGATA<br>CCAGACAGGTTCTCCGGGAGTGGTTCTGGAACGGATTTCACTCTGACGA<br>TCTCACGTTTGGAGCCGGAAGACTTCGCTGTATACTACTGCCAGCAATAT<br>GGATCCTTGCCGTGGACATTCGGGCAAGGAACCAAGGTTGAGATCAAGA<br>GAGGCGGCGGTGGGTCAGGTGGGGGTGGCTCAGGAGGGGGAGGAAGT<br>GGCGGTGGAGGCAGTGAAGTACAGTTAGTCGAGTCCGGAGGTGGGTTA<br>GTACAACCCGGTGGTAGCTTACGTTTATCATGCGCCGCTTCTGGGTTTAC<br>GTTCAGTAGATACTGGATGTCTTGGGTACGTCAGGCGCCCGGAAAAGGG<br>CTGGAGTGGGTAGCAAATATCAAGCAAGATGGATCAGAGAAGTATTATGT<br>CGACTCCGTTAAGGGGAGATTTACAATCTCCCGTGACAATGCCAAGAACA<br>GTTTATATTTACAGATGAATAGCTTAAGAGCTGAGGACACTGCGGTGTATT<br>ATTGTGCGCGTGAAGGGGGCTGGTTTGGAGAGCTGGCGTTTGACTACTG<br>GGGTCAAGGGACTCTTGTGACTGTCTCAAGT
Avelumab scFv geneblock	GCTAGCATGACTGGTGGACAGCAAATGGGTCGGGATCTGTACGACGATG<br>ACGATAAGGTACCAGGATCCAGTCAGTCCGCATTAACACAGCCTGCCTCC<br>GTCAGCGGATCACCGGGGCAGTCCATAACCATCTCCTGCACAGGTACTA<br>GCAGTGACGTTGGTGGCTACAATTACGTCTCCTGGTACCAGCAGCACCC

	CGGGAAAGCCCCCAAGCTTATGATATATGATGTGTCCAACAGACCATCAG<br>GTGTAAGCAACAGGTTTAGTGGAAGCAAATCCGGAAACACTGCGTCTTTG<br>ACGATAAGCGGACTACAAGCGGAGGATGAGGCTGACTACTATTGTTCAAG<br>CTATACTAGTTCTTCTACCAGGGTCTTCGGTACAGGGACGAAAGTAACCG<br>TCTTGGGAGGTGGGGGCTCTGGTGGTGGAGGAAGTGGCGGGGGAGGT<br>AGCGGGGGCGGCGGATCTGAGGTTCAATTGCTTGAATCCGGCGGAGGG<br>CTAGTTCAGCCAGGGGGTAGCCTTAGACTATCTTGCGCTGCGAGTGGGT<br>TCACTTTCTCTTCATATATTATGATGTGGGTGCGTCAGGCGCCCGGAAAG<br>GGATTGGAGTGGGTCTCAAGTATCTACCCCAGCGGGGGGATCACATTTTA<br>CGCCGATACGGTCAAGGGCCGTTTTACCATATCCAGAGACAATAGCAAGA<br>ACACGCTGTATTTACAAATGAATTCTCTAAGGGCTGAAGATACCGCAGTAT<br>ACTATTGTGCCAGGATCAAACTTGGTACCGTAACAACCGTTGATTATTGGG<br>GTCAGGGAACTCTAGTTACAGTATCTTCT

Supplementary Table 3. The amino acid sequences of the 21 mutants selected for further experimental evaluation.

In original dataset?/Mutant ID Number	Mutations	Heavy Chain Sequence
No, 1	K[43]Q,A[79]T,A[97]V,R[98]C	EVQLVESGGGLVQPGGSLRLSCAASGFTFSDSWIHWVRQAPGQGLEWVAWISPYGGSTYYADSVKGRFTISADTSKNTTYLQMNSLRAEDTAVYYCVCRHWPGGFDYWGQGTLVTVSS
No, 2	K[43]Q,I[70]A,A[79]T,A[97]V,R[98]C	EVQLVESGGGLVQPGGSLRLSCAASGFTFSDSWIHWVRQAPGQGLEWVAWISPYGGSTYYADSVKGRFTASADTSKNTTYLQMNSLRAEDTAVYYCVCRHWPGGFDYWGQGTLVTVSS
No, 3	K[43]R,I[70]T,A[79]T,A[97]V	EVQLVESGGGLVQPGGSLRLSCAASGFTFSDSWIHWVRQAPGRGLEWVAWISPYGGSTYYADSVKGRFTTSADTSKNTTYLQMNSLRAEDTAVYYCVRRHWPGGFDYWGQGTLVTVSS
No, 4	I[70]A,A[79]T,A[97]V	EVQLVESGGGLVQPGGSLRLSCAASGFTFSDSWIHWVRQAPGKGLEWVAWISPYGGSTYYADSVKGRFTASADTSKNTTYLQMNSLRAEDTAVYYCVRRHWPGGFDYWGQGTLVTVSS
No, 5	K[43]E,T[58]A,I[70]T,A[79]T,A[97]V,R[98]C	EVQLVESGGGLVQPGGSLRLSCAASGFTFSDSWIHWVRQAPGEGLEWVAWISPYGGSAYYADSVKGRFTTSADTSKNTTYLQMNSLRAEDTAVYYCVCRHWPGGFDYWGQGTLVTVSS
No, 6	K[43]R,T[58]A,I[70]T,A[79]T,A[97]V,R[98]C	EVQLVESGGGLVQPGGSLRLSCAASGFTFSDSWIHWVRQAPGRGLEWVAWISPYGGSAYYADSVKGRFTTSADTSKNTTYLQMNSLRAEDTAVYYCVCRHWPGGFDYWGQGTLVTVSS
No, 7	I[70]A,A[79]T,A[97]V,R[98]C	EVQLVESGGGLVQPGGSLRLSCAASGFTFSDSWIHWVRQAPGKGLEWVAWISPYGGSTYYADSVKGRFTASADTSKNTTYLQMNSLRAEDTAVYYCVCRHWPGGFDYWGQGTLVTVSS
No, 8	T[58]A,I[70]T,A[79]T,A[97]V,R[98]C	EVQLVESGGGLVQPGGSLRLSCAASGFTFSDSWIHWVRQAPGKGLEWVAWISPYGGSAYYADSVKGRFTTSADTSKNTTYLQMNSLRAEDTAVYYCVCRHWPGGFDYWGQGTLVTVSS
No, 9	T[58]A,N[77]S,A[79]V,A[97]V,R[98]C	EVQLVESGGGLVQPGGSLRLSCAASGFTFSDSWIHWVRQAPGKGLEWVAWISPYGGSAYYADSVKGRFTISADTSKSTVYLQMNSLRAEDTAVYYCVCRHWPGGFDYWGQGTLVTVSS

No, 10	T[58]A,N[77]D,A[79]V,A[97]V, R[98]C	EVQLVESGGGLVQPGGSLRLSCAASGFTFSDSWIH WVRQAPGKGLEWVAWISPYGGSAYYADSVKGRFTI SADTSKDTVYLQMNSLRAEDTAVYYCVCRHWPGG FDYWGQGTLVTVSS
No, 11	A[40]T,K[43]R,T[58]A,N[77]D, A[79]T,R[98]C	EVQLVESGGGLVQPGGSLRLSCAASGFTFSDSWIH WVRQTPGRGLEWVAWISPYGGSAYYADSVKGRFTI SADTSKDTTYLQMNSLRAEDTAVYYCACRHWPGGF DYWGQGTLVTVSS
No, 12	A[40]T,K[43]E,T[58]A,N[77]D, A[79]T,A[97]T,R[98]C	EVQLVESGGGLVQPGGSLRLSCAASGFTFSDSWIH WVRQTPGEGLEWVAWISPYGGSAYYADSVKGRFTI SADTSKDTTYLQMNSLRAEDTAVYYCTCRHWPGGF DYWGQGTLVTVSS
No ,13	A[40]T,K[43]R,T[58]A,I[70]A,N[77]D,A[79]T,A[97]V,R[98]C	EVQLVESGGGLVQPGGSLRLSCAASGFTFSDSWIH WVRQTPGRGLEWVAWISPYGGSAYYADSVKGRFT ASADTSKDTTYLQMNSLRAEDTAVYYCVCRHWPG GFDYWGQGTLVTVSS
No, 14	A[40]V,K[43]R,T[58]A,I[70]A,N[77]D,A[79]T,A[97]V,R[98]C	EVQLVESGGGLVQPGGSLRLSCAASGFTFSDSWIH WVRQVPGRGLEWVAWISPYGGSAYYADSVKGRFT ASADTSKDTTYLQMNSLRAEDTAVYYCVCRHWPG GFDYWGQGTLVTVSS
No, 15	A[40]V,T[58]A,I[70]A,N[77]D,A[79]T,S[85]T,A[97]V,R[98]C	EVQLVESGGGLVQPGGSLRLSCAASGFTFSDSWIH WVRQVPGKGLEWVAWISPYGGSAYYADSVKGRFT ASADTSKDTTYLQMNTLRAEDTAVYYCVCRHWPGG FDYWGQGTLVTVSS
No, 16	A[40]V,T[58]A,I[70]A,N[77]D,A[79]I,A[97]V,R[98]C	EVQLVESGGGLVQPGGSLRLSCAASGFTFSDSWIH WVRQVPGKGLEWVAWISPYGGSAYYADSVKGRFT ASADTSKDTIYLQMNSLRAEDTAVYYCVCRHWPGG FDYWGQGTLVTVSS
No, 17	A[40]T,T[58]A,I[70]T,N[77]D,A[79]T,A[97]V,R[98]C	EVQLVESGGGLVQPGGSLRLSCAASGFTFSDSWIH WVRQTPGKGLEWVAWISPYGGSAYYADSVKGRFT TSADTSKDTTYLQMNSLRAEDTAVYYCVCRHWPGG FDYWGQGTLVTVSS
No, 18	A[40]V,T[58]A,I[70]T,N[77]D,A[79]T,A[97]V,R[98]C	EVQLVESGGGLVQPGGSLRLSCAASGFTFSDSWIH WVRQVPGKGLEWVAWISPYGGSAYYADSVKGRFT TSADTSKDTTYLQMNSLRAEDTAVYYCVCRHWPGG FDYWGQGTLVTVSS
No, 19	I[70]V,N[77]D,A[79]T,A[97]V	EVQLVESGGGLVQPGGSLRLSCAASGFTFSDSWIH WVRQAPGKGLEWVAWISPYGGSTYYADSVKGRFT VSADTSKDTTYLQMNSLRAEDTAVYYCVRRHWPG GFDYWGQGTLVTVSS
No, 20	I[70]A,N[77]D,A[79]T,A[97]V	EVQLVESGGGLVQPGGSLRLSCAASGFTFSDSWIH WVRQAPGKGLEWVAWISPYGGSTYYADSVKGRFT ASADTSKDTTYLQMNSLRAEDTAVYYCVRRHWPG

		GFDYWGQGTLVTVSS
No, 21	T[58]A, I[70]T, N[77]D, S[85]F, R[98]C	EVQLVESGGGLVQPGGSLRLSCAASGFTFSDSWIH WVRQAPGKGLEWVAWISPYGGSAYYADSVKGRFT TSADTSKDTAYLQMNFLRAEDTAVYYCACRHWPGG FDYWGQGTLVTVSS

Supplementary Table 4. Change in affinity model-assigned score for each mutation found in the final 21 selected sequences, if that mutation were introduced into the wild type with no other modifications

Mutation	Change in score (positive = more likely to be a strong binder)
K[43]Q	0.164
A[79]T	2.941
A[97]V	1.442
R[98]C	5.013
I[70]A	3.351
K[43]R	0.013
I[70]T	1.677
K[43]E	-0.050
T[58]A	2.125
N[77]S	0.074
A[79]V	0.215
N[77]D	0.839
A[40]T	-0.004
A[97]T	-0.047
A[40]V	-0.007
S[85]T	0.026
A[79]I	5.071
I[70]V	0.142
S[85]F	-0.074

Supplementary Table 5. Change in affinity model assigned score (positive = more likely to be a strong binder) if removing the specified mutation from the specified sequence selected for experimental evaluation

Mutation	Sequence	Score_shift
K[43]Q	K[43]Q_A[79]T_A[97]V_R[98]C	-1.350174139
K[43]Q	K[43]Q_I[70]A_A[79]T_A[97]V_R[98]C	-1.199340395
A[79]T	K[43]Q_A[79]T_A[97]V_R[98]C	-2.90800495
A[79]T	K[43]Q_I[70]A_A[79]T_A[97]V_R[98]C	-2.860648684
A[79]T	K[43]R_I[70]T_A[79]T_A[97]V	-3.767419545
A[79]T	I[70]A_A[79]T_A[97]V	-3.641706143
A[79]T	K[43]E_T[58]A_I[70]T_A[79]T_A[97]V_R[98]C	-2.459017111
A[79]T	K[43]R_T[58]A_I[70]T_A[79]T_A[97]V_R[98]C	-2.515520463
A[79]T	I[70]A_A[79]T_A[97]V_R[98]C	-2.661562361
A[79]T	T[58]A_I[70]T_A[79]T_A[97]V_R[98]C	-2.60147677
A[79]T	A[40]T_K[43]R_T[58]A_N[77]D_A[79]T_R[98]C	-2.245035507
A[79]T	A[40]T_K[43]E_T[58]A_N[77]D_A[79]T_A[97]T_R[98]C	-2.353740979
A[79]T	A[40]T_K[43]R_T[58]A_I[70]A_N[77]D_A[79]T_A[97]V_R[98]C	-2.528502451
A[79]T	A[40]V_K[43]R_T[58]A_I[70]A_N[77]D_A[79]T_A[97]V_R[98]C	-2.515708944
A[79]T	A[40]V_T[58]A_I[70]A_N[77]D_A[79]T_S[85]T_A[97]V_R[98]C	-2.598529857
A[79]T	A[40]T_T[58]A_I[70]T_N[77]D_A[79]T_A[97]V_R[98]C	-2.737110257
A[79]T	A[40]V_T[58]A_I[70]T_N[77]D_A[79]T_A[97]V_R[98]C	-2.734786899
A[79]T	I[70]V_N[77]D_A[79]T_A[97]V	-4.011675647
A[79]T	I[70]A_N[77]D_A[79]T_A[97]V	-2.933384354
A[97]V	K[43]Q_A[79]T_A[97]V_R[98]C	-3.258461187
A[97]V	K[43]Q_I[70]A_A[79]T_A[97]V_R	-3.214925341

	[98]C	
A[97]V	K[43]R_I[70]T_A[79]T_A[97]V	-3.613088338
A[97]V	I[70]A_A[79]T_A[97]V	-4.113650952
A[97]V	K[43]E_T[58]A_I[70]T_A[79]T_A[97]V_R[98]C	-3.45793183
A[97]V	K[43]R_T[58]A_I[70]T_A[79]T_A[97]V_R[98]C	-3.070166001
A[97]V	I[70]A_A[79]T_A[97]V_R[98]C	-3.035787977
A[97]V	T[58]A_I[70]T_A[79]T_A[97]V_R[98]C	-3.019094568
A[97]V	T[58]A_N[77]S_A[79]V_A[97]V_R[98]C	-3.251001503
A[97]V	T[58]A_N[77]D_A[79]V_A[97]V_R[98]C	-3.144622633
A[97]V	A[40]T_K[43]R_T[58]A_I[70]A_N[77]D_A[79]T_A[97]V_R[98]C	-3.068949686
A[97]V	A[40]V_K[43]R_T[58]A_I[70]A_N[77]D_A[79]T_A[97]V_R[98]C	-3.072628042
A[97]V	A[40]V_T[58]A_I[70]A_N[77]D_A[79]T_S[85]T_A[97]V_R[98]C	-3.071779292
A[97]V	A[40]V_T[58]A_I[70]A_N[77]D_A[79]I_A[97]V_R[98]C	-3.077552587
A[97]V	A[40]T_T[58]A_I[70]T_N[77]D_A[79]T_A[97]V_R[98]C	-3.069431424
A[97]V	A[40]V_T[58]A_I[70]T_N[77]D_A[79]T_A[97]V_R[98]C	-3.072235019
A[97]V	I[70]V_N[77]D_A[79]T_A[97]V	-4.14747887
A[97]V	I[70]A_N[77]D_A[79]T_A[97]V	-4.396574433
R[98]C	K[43]Q_A[79]T_A[97]V_R[98]C	-4.681981752
R[98]C	K[43]Q_I[70]A_A[79]T_A[97]V_R[98]C	-4.564467958
R[98]C	K[43]E_T[58]A_I[70]T_A[79]T_A[97]V_R[98]C	-4.482899024
R[98]C	K[43]R_T[58]A_I[70]T_A[79]T_A[97]V_R[98]C	-3.984356293
R[98]C	I[70]A_A[79]T_A[97]V_R[98]C	-4.065552153
R[98]C	T[58]A_I[70]T_A[79]T_A[97]V_R[98]C	-4.032531839

R[98]C	T[58]A_N[77]S_A[79]V_A[97]V_R[98]C	-4.053472664
R[98]C	T[58]A_N[77]D_A[79]V_A[97]V_R[98]C	-3.925880262
R[98]C	A[40]T_K[43]R_T[58]A_N[77]D_A[79]T_R[98]C	-3.792805053
R[98]C	A[40]T_K[43]E_T[58]A_N[77]D_A[79]T_A[97]T_R[98]C	-3.808925915
R[98]C	A[40]T_K[43]R_T[58]A_I[70]A_N[77]D_A[79]T_A[97]V_R[98]C	-3.459664332
R[98]C	A[40]V_K[43]R_T[58]A_I[70]A_N[77]D_A[79]T_A[97]V_R[98]C	-3.445673009
R[98]C	A[40]V_T[58]A_I[70]A_N[77]D_A[79]T_S[85]T_A[97]V_R[98]C	-3.663700145
R[98]C	A[40]V_T[58]A_I[70]A_N[77]D_A[79]I_A[97]V_R[98]C	-3.333244115
R[98]C	A[40]T_T[58]A_I[70]T_N[77]D_A[79]T_A[97]V_R[98]C	-3.400800824
R[98]C	A[40]V_T[58]A_I[70]T_N[77]D_A[79]T_A[97]V_R[98]C	-3.399711521
R[98]C	T[58]A_I[70]T_N[77]D_S[85]F_R[98]C	-4.637969063
I[70]A	K[43]Q_I[70]A_A[79]T_A[97]V_R[98]C	-3.956632189
I[70]A	I[70]A_A[79]T_A[97]V	-4.622633133
I[70]A	I[70]A_A[79]T_A[97]V_R[98]C	-4.107466139
I[70]A	A[40]T_K[43]R_T[58]A_I[70]A_N[77]D_A[79]T_A[97]V_R[98]C	-4.104208933
I[70]A	A[40]V_K[43]R_T[58]A_I[70]A_N[77]D_A[79]T_A[97]V_R[98]C	-4.106134435
I[70]A	A[40]V_T[58]A_I[70]A_N[77]D_A[79]T_S[85]T_A[97]V_R[98]C	-4.269910854
I[70]A	A[40]V_T[58]A_I[70]A_N[77]D_A[79]I_A[97]V_R[98]C	-4.201326161
I[70]A	I[70]A_N[77]D_A[79]T_A[97]V	-4.91781276
K[43]R	K[43]R_I[70]T_A[79]T_A[97]V	-0.442899434
K[43]R	K[43]R_T[58]A_I[70]T_A[79]T_A[97]V_R[98]C	-0.216553101
K[43]R	A[40]T_K[43]R_T[58]A_N[77]D_	-0.149536468

	A[79]T_R[98]C	
K[43]R	A[40]T_K[43]R_T[58]A_I[70]A_N[77]D_A[79]T_A[97]V_R[98]C	-0.157092081
K[43]R	A[40]V_K[43]R_T[58]A_I[70]A_N[77]D_A[79]T_A[97]V_R[98]C	-0.098514577
I[70]T	K[43]R_I[70]T_A[79]T_A[97]V	-3.023319451
I[70]T	K[43]E_T[58]A_I[70]T_A[79]T_A[97]V_R[98]C	-2.025705649
I[70]T	K[43]R_T[58]A_I[70]T_A[79]T_A[97]V_R[98]C	-2.064781556
I[70]T	T[58]A_I[70]T_A[79]T_A[97]V_R[98]C	-2.015312296
I[70]T	A[40]T_T[58]A_I[70]T_N[77]D_A[79]T_A[97]V_R[98]C	-1.748111844
I[70]T	A[40]V_T[58]A_I[70]T_N[77]D_A[79]T_A[97]V_R[98]C	-1.749517353
I[70]T	T[58]A_I[70]T_N[77]D_S[85]F_R[98]C	-1.520065354
K[43]E	K[43]E_T[58]A_I[70]T_A[79]T_A[97]V_R[98]C	0.310477899
K[43]E	A[40]T_K[43]E_T[58]A_N[77]D_A[79]T_A[97]T_R[98]C	0.268295001
T[58]A	K[43]E_T[58]A_I[70]T_A[79]T_A[97]V_R[98]C	-2.502012564
T[58]A	K[43]R_T[58]A_I[70]T_A[79]T_A[97]V_R[98]C	-2.545627961
T[58]A	T[58]A_I[70]T_A[79]T_A[97]V_R[98]C	-2.444753748
T[58]A	T[58]A_N[77]S_A[79]V_A[97]V_R[98]C	-2.481147911
T[58]A	T[58]A_N[77]D_A[79]V_A[97]V_R[98]C	-2.470916578
T[58]A	A[40]T_K[43]R_T[58]A_N[77]D_A[79]T_R[98]C	-2.560322143
T[58]A	A[40]T_K[43]E_T[58]A_N[77]D_A[79]T_A[97]T_R[98]C	-2.517952252
T[58]A	A[40]T_K[43]R_T[58]A_I[70]A_N[77]D_A[79]T_A[97]V_R[98]C	-2.553986536
T[58]A	A[40]V_K[43]R_T[58]A_I[70]A_N	-2.554008504

	[77]D_A[79]T_A[97]V_R[98]C	
T[58]A	A[40]V_T[58]A_I[70]A_N[77]D_A[79]T_S[85]T_A[97]V_R[98]C	-2.501791042
T[58]A	A[40]V_T[58]A_I[70]A_N[77]D_A[79]I_A[97]V_R[98]C	-2.498084813
T[58]A	A[40]T_T[58]A_I[70]T_N[77]D_A[79]T_A[97]V_R[98]C	-2.488346219
T[58]A	A[40]V_T[58]A_I[70]T_N[77]D_A[79]T_A[97]V_R[98]C	-2.487205417
T[58]A	T[58]A_I[70]T_N[77]D_S[85]F_R[98]C	-2.246574448
N[77]S	T[58]A_N[77]S_A[79]V_A[97]V_R[98]C	-1.441761162
A[79]V	T[58]A_N[77]S_A[79]V_A[97]V_R[98]C	-0.708267357
A[79]V	T[58]A_N[77]D_A[79]V_A[97]V_R[98]C	-1.030471632
N[77]D	T[58]A_N[77]D_A[79]V_A[97]V_R[98]C	-2.732021162
N[77]D	A[40]T_K[43]R_T[58]A_N[77]D_A[79]T_R[98]C	-1.845184662
N[77]D	A[40]T_K[43]E_T[58]A_N[77]D_A[79]T_A[97]T_R[98]C	-1.847152997
N[77]D	A[40]T_K[43]R_T[58]A_I[70]A_N[77]D_A[79]T_A[97]V_R[98]C	-1.905583369
N[77]D	A[40]V_K[43]R_T[58]A_I[70]A_N[77]D_A[79]T_A[97]V_R[98]C	-1.895088216
N[77]D	A[40]V_T[58]A_I[70]A_N[77]D_A[79]T_S[85]T_A[97]V_R[98]C	-2.240900081
N[77]D	A[40]V_T[58]A_I[70]A_N[77]D_A[79]I_A[97]V_R[98]C	-1.855594426
N[77]D	A[40]T_T[58]A_I[70]T_N[77]D_A[79]T_A[97]V_R[98]C	-1.526277661
N[77]D	A[40]V_T[58]A_I[70]T_N[77]D_A[79]T_A[97]V_R[98]C	-1.52679816
N[77]D	I[70]V_N[77]D_A[79]T_A[97]V	-1.796762756
N[77]D	I[70]A_N[77]D_A[79]T_A[97]V	-2.510237153
N[77]D	T[58]A_I[70]T_N[77]D_S[85]F_R[98]C	-1.322139786

A[40]T	A[40]T_K[43]R_T[58]A_N[77]D_A[79]T_R[98]C	-0.985338547
A[40]T	A[40]T_K[43]E_T[58]A_N[77]D_A[79]T_A[97]T_R[98]C	-1.04431944
A[40]T	A[40]T_K[43]R_T[58]A_I[70]A_N[77]D_A[79]T_A[97]V_R[98]C	-0.985222803
A[40]T	A[40]T_T[58]A_I[70]T_N[77]D_A[79]T_A[97]V_R[98]C	-0.993360638
A[97]T	A[40]T_K[43]E_T[58]A_N[77]D_A[79]T_A[97]T_R[98]C	-0.564682294
A[40]V	A[40]V_K[43]R_T[58]A_I[70]A_N[77]D_A[79]T_A[97]V_R[98]C	-0.986005803
A[40]V	A[40]V_T[58]A_I[70]A_N[77]D_A[79]T_S[85]T_A[97]V_R[98]C	-1.055614513
A[40]V	A[40]V_T[58]A_I[70]A_N[77]D_A[79]I_A[97]V_R[98]C	-1.080123693
A[40]V	A[40]V_T[58]A_I[70]T_N[77]D_A[79]T_A[97]V_R[98]C	-1.052150638
S[85]T	A[40]V_T[58]A_I[70]A_N[77]D_A[79]T_S[85]T_A[97]V_R[98]C	-0.292705577
A[79]I	A[40]V_T[58]A_I[70]A_N[77]D_A[79]I_A[97]V_R[98]C	-4.115737706
I[70]V	I[70]V_N[77]D_A[79]T_A[97]V	-1.39518576
S[85]F	T[58]A_I[70]T_N[77]D_S[85]F_R[98]C	0.384279205

SUPPLEMENTARY METHODS

S2.1 Sequence Quality

Sequence read pairs that contained one or more base pairs with a phred quality score < 10 were discarded since the sequence read in this case may be unreliable. In the event the overlapping region of the paired ends did not match, both reads were discarded, so that no mismatches between the paired ends were allowed. The reads that met these quality criteria were merged and translated to yield mutant atezolizumab sequences of 118 amino acids in length. Each sequence that occurred more than once in any given category was assigned a frequency for that category indicating the number of times it was found. Sequences with mutations in the first 30 or last 8 positions were excluded from further consideration, since these positions were not targeted for mutation and mutations at these positions were very rare so it was difficult to assess their importance.

S2.2 Generation of the WT and Mutant Atezolizumab scFv Library

To test the function of the WT scFv in the yeast display format, the gene for WT Atezolizumab scFv[1] was purchased as a geneblock (using codons optimized for yeast) from Integrated DNA Technologies and cloned into a modified pYD1(Addgene #73447) yeast display vector by PCR amplification of the geneblock with AtezF/AtezR primers (Supplemental Table S2) and then using double digestion/ligation into a digested vector by Golden Gate Assembly[2,3]. (Esp3I (Thermo Scientific) and T4 DNA Ligase (New England Biolabs (NEB))) and sequence verified before transformation into EBY100 yeasts via [4]. For the Atezolizumab library, the WT scFv in the pYD1 vector was used as template for either high fidelity PCR of the light chain region with Q5 hotstart DNA polymerase (NEB) (primers IF1F/IF1R) or error-prone PCR of the heavy chain region with Taq DNA polymerase (Invitrogen 18038-018, IF2F/IF2R) using 8-oxo-dGTP (TriLink N-2034-1) and dPTP (TriLink N-2037-1)[5], except 30 cycles of error-prone PCR with 200 μM each dPTP and 8-oxo-dGTP (final concentration 20 μM each) were used to increase the mutation rate. The PCR products of the light/heavy chains were concentrated by the DNA Clean and Concentrator-5 kit (DCC-5, Zymo Research) and purified by an agarose gel extraction kit (Zymo Research gel extraction kit). The 2 DNA fragments (WT light chain, mutated heavy chain) were assembled into a single DNA molecule by overlap extension PCR using the OF/OR primers (all primers in Table S2) with Q5 hotstart DNA polymerase, the resulting product was again concentrated by DCC-5 kit and purified by gel extraction. TA cloning (Invitrogen TA Cloning Kit, K202020) by adding 3'A to the product with Taq was used to examine the mutation rate in the heavy chain, random sequencing of clones revealed 0-8 mutations per gene. In preparation for yeast electroporation, the pYD1 vector was double digested with XhoI and EcoRI-HF, concentrated by DCC-5 kit, and gel extracted/eluted into ddH20.

The scFv library was transformed into yeast by electroporation (3 μg digested pYD1, 9 μg scFv gene per electroporation) and assembled in the EBY100 cells (ATCC MYA-4941) by homologous recombination[6], giving 7.8 x 10^7 transformants as determined by serial dilution onto plates with selective growth media (16.7 g/L BD bactoagar for solid media (see [7] for more on media recipe)). The library was passaged several times in selective growth media to ensure 1 plasmid/cell before being frozen in aliquots at -80°C in 85% ddH20, 10% glycerol, 5% DMSO (3.6 x 10^8 cells/tube). As a control for sorting, the WT scFv gene/pYD1 was also electroporated into EBY100 so as to be in the exact same position in the vector as the library.

S2.3 Atezolizumab scFv Library Screening by Yeast Surface Display

First, in order to determine the optimal competition time, the WT scFv koff (off-rate) towards PD-L1 was determined basically as previously described[8]. Yeasts expressing the WT scFv were washed with TBS-BSA and resuspended to 16 x 10^6 cells in 400 uL volume. Then, 12 uL b-PDL1 (final concentration 178 nM, Sino Biological 10084-H08H-B) was added to the yeasts and labeled at RT for 3hrs. Afterwards, the yeasts were washed with TBS-BSA and resuspended to 800 uL total volume with 174 nM unlabeled PDL1 (Sino Biological 10084-H08H). 2 x 10^6 cells were added to each eppendorf tube and rotated at RT for x hrs (0-21 hrs). For each timepoint, the cells were washed 3x with TBS-BSA (cold) and kept on ice in 198 uL TBS-BSA until the experiment was completed. Once all data points were collected, 1:100 V5 antibody (R960-25, previously known as 46-0705) was added and the yeasts labeled on ice for 1hr. The yeasts were washed with TBS-BSA (cold) and resuspended in 200 uL with 1:100 SA-PE (BD 554061) and goat anti-mouse AF647 (Thermo Fisher A21241) and labeled on ice for 30 minutes. Afterwards the yeasts were washed with TBS-BSA (cold) and resuspended in TBS-BSA (cold) for flow cytometry. The optimal competition time was determined to be 29 hours at RT[9]. To screen the library against PD-L1, either the WT or library was thawed and added to selective growth media (20 x 10^6 cells into 50 mL growth media + ampicillin, grown with shaking at 30°C for 22 hrs, followed by induction by pelleting the cells, washing them once with sterile ddH20, resuspension into the same type of media but with 20 g/L galactose instead of glucose, pH adjusted to 5.6 and OD_{600} = 0.2 with ampicillin). The scFv were induced for 42 hr at 20°C with shaking. After 42 hours, the flasks were placed on ice and 40 x 10^6 yeasts (WT or library) were pelleted at 17,000xG for 30s, resuspended in 1 mL TBS-BSA (25 mM Tris, 150 mM NaCl, pH 7.5, 5 mg/mL BSA), re-pelleted, resuspended in TBS-BSA buffer at 40 million cells/mL. WT or library was then labeled with biotin-PD-L1 (Sino Biological 10084-H08H-B) at a final concentration of 178 nM for 3 hrs at RT with rotation. Afterwards, the cells were pelleted/washed with TBS-BSA, then resuspended in non-biotin PD-L1 (Sino Biological 10084-H08H, 174 nM) and rotated for various amounts of time (mostly 29 hours, but one screen was for 7 hours) at RT. For sorting by FACS, cells were pelleted/washed 3x with cold TBS-BSA and labeled with anti-V5 antibody (Thermo Fisher R960-25, validated for specificity by the vendor, lots 2001339/2249078/2212258) at 1:100 dilution on ice for 60 minutes (at 10^7 cells/mL), pelleted/washed with cold buffer, then labeled with 1:100 each secondary detection reagent (PE Streptavidin BD 554061, goat anti-mouse IgG2a AF647 (Thermo Fisher A21241) on ice for 30 minutes at 10^7 cells/mL. Cells were then pelleted/washed with cold buffer and resuspended in cold TBS-BSA buffer for sorting on a BD FACSAria II (Moores Cancer Center, UC San Diego) using BD FACSDiva v. 8.0.1 software, see Figure S12 for example FACS plot. Sort gates were set to collect mutants with faster, WT-level, or slower off-rates to PD-L1, see Figure S12 for example of gating. Hits were collected in selective growth media and grown up to high density at 30°C and aliquoted to frozen stocks at -80°C. The process was repeated twice for binders with faster or WT-level off-rates and four times for the mutants with slower off-rates.

S2.4 Generation and Screening of the Focused 21-Mutant Library

Twenty-one geneblocks coding the 21 heavy chain mutants were purchased from IDT, followed by PCR amplification and overlap extension PCR (Q5 Hotstart DNA Polymerase) to generate 21 full-length Atezolizumab scFv mutant genes. The sequences of the 21 heavy chain mutants appear in Table S3 of the Supplementary Info. The genes were electroporated (along with linearized pYD1) into EBY100 yeasts to assemble the focused library and the library was

passaged/frozen as described for the initial library. Titration onto selective media plates revealed >10^7 transformants.

In order to screen the library for the most improved (slowest off-rate) mutants, increasingly long competition times were used in the presence of excess unlabelled PD-L1 (after initially saturating the WT or mutant library with 200 nM biotin-PD-L1, the cells were pelleted/washed to remove unbound biotin-PD-L1 and resuspended in excess (128 nM unlabelled PD-L1) for 7.5h, 17h, then 39h at RT). The most intense clones were collected by FACS (using the WT scFv as a reference) in growth media and propagated to the next round of sorting. After 3 rounds of selection, plasmids from the hits were harvested by yeast miniprep and transformed into GC10 competent cells, which were picked as individual colonies and subject to bacterial miniprep. Plasmids were sequenced using Eton Bioscience sequencing services.

S2.5 Determination of the WT/Mutant 4/Durvalumab/Avelumab K_D and k_{off} Values on the Surface of Yeast

The RT K_{off} (off-rate) of the scFv-PD-L1 complex was determined essentially as described[8] using biotin/non-biotin PD-L1 (same versions used during library screening), with TBS-BSA as the buffer and the same antibody reagents used for library screening (also see section S2.3). The yeasts were grown/induced in the same manner as with the screening protocol and labeled with 200 nM b-PD-L1 at 4 x 10^7 cells/mL, pelleted/washed, followed by 64 nM unlabelled PD-L1 (at 10^7/mL) and rotated at RT. The mean fluorescence intensity (MFI) of the V5 positive yeasts was recorded at each time point and the data fit to a one phase decay model with GraphPad Prism 9.3.0 software ($Y = (Y0 - Plateau)*exp(-K*X) + Plateau$, Y is fraction of yeast bound to biotin-PDL1, X is time, Y0 = 1 (fraction of yeast bound to biotin-PDL1 at the 0s competition time point), plateau is a constant based on nonspecifically bound yeast, and K is Koff). The K_D on the yeast surface was determined as previously described[5], where the MFI of the V5+ population was plotted vs. the antigen concentration and the biotin-PD-L1 the same as used for the library screening. Important notes on the K_D determination include using 10^5 cells per data point, using 1:200 anti-V5 antibody after resuspension of each data point in 100 uL TBS-BSA (cold) for 1hr, washing cells with cold TBS-BSA after V5 mAb incubation, then labeling cells with 1:100 SA-PE/IgG-AF647 for 20 minutes on ice, and washing cells in cold TBS-BSA and resuspension in cold TBS-BSA for flow cytometry. The data was fit to the following equation: $y = B_{max}*X/(K_D+X)$ (Bmax was maximum MFI value, X the antigen concentration, the MFI without PD-L1 was subtracted from all values). Due to the very slow off-rate of Mutant 4, it was necessary to incubate the samples at RT for 6 days to allow the system to get to equilibrium.

S2.6 Cloning and Purification of WT/Mutant 4 Atezolizumab scFv

The genes encoding the scFv sequences were amplified by PCR out of the pYD1 vector with primers containing NcoI-HF and XhoI cut sites (scFv F/R, Table S2) using Q5 hotstart DNA polymerase (NEB). The PCR products were double digested (along with the pET27b(+) vector (69863-3, MilliporeSigma) (the vector was also dephosphorylated with Quick CIP (NEB)). The products of the digestion were ligated using T4 DNA Ligase (NEB) and the ligation transformed into NEB 5-alpha cells. The plasmids were purified using the Zippy Plasmid Miniprep Kit and sequence verified.

The scFv plasmids were transformed into Rosetta(DE3) cells (70954-3, MilliporeSigma) onto LB-0.5% glucose plates with 30 μg/mL kanamycin, 34 μg/mL chloramphenicol and grown in LB-0.5% glucose media supplemented with kanamycin/chloramphenicol (30 μg/mL kanamycin, 34 μg/mL chloramphenicol) (10 mL) for 5 hours at 37°C with shaking until OD_{600} = 0.8-1. Afterwards, 4 mL of the media was added to 400 mL autoinduction media (71300-4, MilliporeSigma, with 100 μg/mL kanamycin and 34 μg/mL chloramphenicol) and shaken at 200 RPM at 25°C for 24 hr with added antibiotics. Afterwards, the scFv-His_6 fusions were purified essentially as described[10], except 5 mM $MgSO_4$ was used instead of $MgSO_2$, supernatant was mixed with 0.3M NaCl (not 0.5M), 5 mL of Ni-NTA agarose (Qiagen 30210) was used instead of 0.5 mL, and protein was exchanged into PBS buffer (140 mM NaCl, 2.7 mM KCl, 10 mM Na_2HPO_4, 1.8 mM KH_2PO_4, pH7.4) after elution using Vivaspin-20 (10,000 MWCO, Cytiva 28932360) filters by centrifugation. Also, the Ni-NTA agarose resin was washed with 50 mM HEPES, 300 mM NaCl, 50 mM imidazole, pH 7.5, then the same buffer but with 70 mM imidazole, followed by elution with the same buffer but with 250 mM imidazole. The concentrated scFv was diluted so as to have 10% glycerol and flash frozen and stored at -80°C. Protein purity was assessed by SDS-PAGE (Figure S5) and concentration determined by BCA assay.

Supplementary References

1. Tan, S. *et al.* Distinct PD-L1 binding characteristics of therapeutic monoclonal antibody durvalumab. *Protein Cell* **9**, 135–139 (2018).
2. Engler, C., Kandzia, R. & Marillonnet, S. A one pot, one step, precision cloning method with high throughput capability. *PloS One* **3**, e3647 (2008).
3. Engler, C., Gruetzner, R., Kandzia, R. & Marillonnet, S. Golden Gate Shuffling: A One-Pot DNA Shuffling Method Based on Type IIs Restriction Enzymes. *PLOS ONE* **4**, e5553 (2009).
4. Gietz, R. D. & Schiestl, R. H. High-efficiency yeast transformation using the LiAc/SS carrier DNA/PEG method. *Nat. Protoc.* **2**, 31–34 (2007).
5. Chao, G. *et al.* Isolating and engineering human antibodies using yeast surface display. *Nat. Protoc.* **1**, 755–768 (2006).
6. Bidlingmaier, S., Su, Y. & Liu, B. Combining Phage and Yeast Cell Surface Antibody Display to Identify Novel Cell Type-Selective Internalizing Human Monoclonal Antibodies. *Methods Mol. Biol. Clifton NJ* **1319**, 51–63 (2015).
7. Parkinson, J., Hard, R., Ainsworth, R. I., Li, N. & Wang, W. Engineering a Histone Reader Protein by Combining Directed Evolution, Sequencing, and Neural Network Based Ordinal Regression. *J. Chem. Inf. Model.* **60**, 3992–4004 (2020).
8. Tillotson, B. J., Lajoie, J. M. & Shusta, E. V. Yeast Display-Based Antibody Affinity Maturation Using Detergent-Solubilized Cell Lysates. *Methods Mol. Biol. Clifton NJ* **1319**, 65–78 (2015).
9. Boder, E. T. & Wittrup, K. D. Optimal Screening of Surface-Displayed Polypeptide Libraries. *Biotechnol. Prog.* **14**, 55–62 (1998).
10. Miller, K. D., Weaver-Feldhaus, J., Gray, S. A., Siegel, R. W. & Feldhaus, M. J. Production, purification, and characterization of human scFv antibodies expressed in Saccharomyces

cerevisiae, Pichia pastoris, and Escherichia coli. *Protein Expr. Purif.* **42**, 255–267 (2005).
