# Supplementary material for: The RESP AI model accelerates the identification of tight-binding antibodies
Source: Nat Commun. 2023 Jan 28;14:454. doi: 10.1038/s41467-023-36028-8 (PMC9884274; doi:10.1038/s41467-023-36028-8)
Supplement: Supplementary file 2 — Reporting Summary [file 41467_2023_36028_MOESM2_ESM.pdf]

nature portfolio

Corresponding author(s): Wei Wang

Last updated by author(s): Jan 6, 2023

Reporting Summary

Nature Portfolio wishes to improve the reproducibility of the work that we publish. This form provides structure for consistency and transparency in reporting. For further information on Nature Portfolio policies, see our Editorial Policies and the Editorial Policy Checklist.

Statistics

For all statistical analyses, confirm that the following items are present in the figure legend, table legend, main text, or Methods section.

n/a	Confirmed	
☐	☒	The exact sample size (*n*) for each experimental group/condition, given as a discrete number and unit of measurement
☐	☒	A statement on whether measurements were taken from distinct samples or whether the same sample was measured repeatedly
☐	☒	The statistical test(s) used AND whether they are one- or two-sided<br>*Only common tests should be described solely by name; describe more complex techniques in the Methods section.*
☐	☒	A description of all covariates tested
☐	☒	A description of any assumptions or corrections, such as tests of normality and adjustment for multiple comparisons
☐	☒	A full description of the statistical parameters including central tendency (e.g. means) or other basic estimates (e.g. regression coefficient) AND variation (e.g. standard deviation) or associated estimates of uncertainty (e.g. confidence intervals)
☐	☒	For null hypothesis testing, the test statistic (e.g. *F*, *t*, *r*) with confidence intervals, effect sizes, degrees of freedom and *P* value noted<br>*Give P values as exact values whenever suitable.*
☒	☐	For Bayesian analysis, information on the choice of priors and Markov chain Monte Carlo settings
☒	☐	For hierarchical and complex designs, identification of the appropriate level for tests and full reporting of outcomes
☒	☐	Estimates of effect sizes (e.g. Cohen's *d*, Pearson's *r*), indicating how they were calculated

Our web collection on statistics for biologists contains articles on many of the points above.

Software and code

Policy information about availability of computer code

Data collection	For data collection, for mass photometry we used Refeyn DiscoverMP software version 2.3.0. FACS experiments used BD FACSDiva version 8.0.1. Yeast Kd and Koff values were fitted using GraphPad Prism 9.3.0. BLI binding kinetics measurements were obtained using Octed HT Software Version 12.
Data analysis	All custom code used for data analysis is available at https://github.com/Wang-lab-UCSD/RESP; this same link is provided in the manuscript. PyTorch v1.8.1, numpy v1.19.5, scipy v1.5.4 and scikit-learn v0.24.2 were the Python libraries used for model training and data analysis; this same information is provided in the manuscript. Yeast Kd and Koff values were fitted using GraphPad Prism 9.3.0.

For manuscripts utilizing custom algorithms or software that are central to the research but not yet described in published literature, software must be made available to editors and reviewers. We strongly encourage code deposition in a community repository (e.g. GitHub). See the Nature Portfolio guidelines for submitting code & software for further information.

Data

Policy information about availability of data

All manuscripts must include a data availability statement. This statement should provide the following information, where applicable:
- Accession codes, unique identifiers, or web links for publicly available datasets
- A description of any restrictions on data availability
- For clinical datasets or third party data, please ensure that the statement adheres to our policy

The raw sequence read data generated for this study has been uploaded to the Sequence Read Archive (SRA) database under accession code PRJNA813220 [https://

www.ncbi.nlm.nih.gov/bioproject/PRJNA813220/] . The antibody sequence data used to train the autoencoder used in this study are available in the cAbRep database [https://cab-rep.c2b2.columbia.edu/] . The construction of the cAbRep database is described in Guo et al.

Human research participants

Policy information about studies involving human research participants and Sex and Gender in Research.

Reporting on sex and gender	N/A
Population characteristics	N/A
Recruitment	N/A
Ethics oversight	N/A

Note that full information on the approval of the study protocol must also be provided in the manuscript.

Field-specific reporting

Please select the one below that is the best fit for your research. If you are not sure, read the appropriate sections before making your selection.

- [x] Life sciences
- [ ] Behavioural & social sciences
- [ ] Ecological, evolutionary & environmental sciences

For a reference copy of the document with all sections, see nature.com/documents/nr-reporting-summary-flat.pdf

Life sciences study design

All studies must disclose on these points even when the disclosure is negative.

Sample size	No statistical method was used to predetermine sample size. This is because the study describes the construction of a machine learning based pipeline for identifying novel tight-binding sequences, and for machine learning model construction, it is preferable to use as much training data as possible to improve the model's ability to generalize, therefore selecting a subset of the data for training would not be desirable, and thus there is no sample size calculation involved. The test set for comparing performance of different models was constructed by randomly selecting 20% of the assembled sequences and assigning these to test as is typical for machine learning model evaluation. The random partition was generated using the Mersenne Twister random number generation algorithm as implemented in Python’s numpy library version 1.19.5 with a seed value of 0. When performance of different models was compared using cross-validations, the cross-validation splits were generated by randomly partitioning the dataset into 5 splits of equal size using the KFold function in Python’s scikit-learn library version 0.24.2.
Data exclusions	When processing raw sequence data, unreliable sequence reads (reads containing one or more bases with a phred quality score < 10 or where the paired end reads did not match in the overlap region) were discarded before any further analysis or processing was conducted. These steps were taken to ensure that only reliable reads were used for analysis. No data was otherwise excluded from any subsequent analysis or model training.
Replication	The yeast Koff experiment was done twice (see Figure 5B/C). The K D was determined on the yeast surface 3 times independently, the mass photometry of the WT and mutant scFv repeated once with 2 batches of scFv (2 replicates), the SDS-PAGE of purified protein was performed for each new batch of scFv (2 gels, Figure S5). All attempts at replication were successful.
Randomization	Measurements of Kd and koff do not require a randomization method. The K D measurements were made in 3 independent replicates and the Koff was measured using 2 different methods (yeast Koff assay and BLI measurements). Library screening and mass photometry also are methods where randomization is unnecessary. Because none of these techniques require the researcher to randomly assign participants to a group or randomly extract data for analysis this question is not relevant to the experimental data generated in this study.<br><br>When comparing performance of different machine learning models, a subset of the available data was assigned to the test set before any model training was conducted. The test set for comparing performance of different models was constructed by randomly selecting 20% of the assembled sequences and assigning these to test as is typical for machine learning model evaluation. The random partition was generated using the Mersenne Twister random number generation algorithm as implemented in Python’s numpy library version 1.19.5 with a seed value of 0. When performance of different models was compared using cross-validations, the cross-validation splits were generated by randomly partitioning the dataset into 5 splits of equal size using the KFold function in Python’s scikit-learn library version 0.24.2.
Blinding	The performance of the pipeline was evaluated by experimentally testing predictions made by the model for sequences not present in the original dataset and for which no Kd data was available; these are "blind" predictions since the experimental Kd test was performed after the predictions had been made. The procedure by which these predictions were made and tested is described in the manuscript.

Reporting for specific materials, systems and methods

We require information from authors about some types of materials, experimental systems and methods used in many studies. Here, indicate whether each material, system or method listed is relevant to your study. If you are not sure if a list item applies to your research, read the appropriate section before selecting a response.

Materials & experimental systems

n/a	Involved in the study
☐	☒ Antibodies
☐	☒ Eukaryotic cell lines
☒	☐ Palaeontology and archaeology
☒	☐ Animals and other organisms
☒	☐ Clinical data
☒	☐ Dual use research of concern

Methods

n/a	Involved in the study
☒	☐ ChIP-seq
☐	☒ Flow cytometry
☒	☐ MRI-based neuroimaging

Antibodies

Antibodies used	anti-V5 antibody (Thermo Fisher R96025, lots 2378586/2249078/2212258), Streptavidin-PE (BD Bioscience 554061, lots 8277899/1057256/0022113), and goat anti-mouse IgG2a AF647 (Thermo Fisher A21241, lot 2366136).
Validation	The only primary antibody used was the anti-V5 antibody (Thermo Fisher R960-25, lots 2378586/2249078/2212258) for the FACS experiments. The antibody was validated by immunofluorescence and western blot assays on the manufacturer's website (V5 Tag Monoclonal Antibody (R960-25) (thermofisher.com)). The company says "This Antibody was verified by Relative expression to ensure that the antibody binds to the antigen stated."

Eukaryotic cell lines

Policy information about cell lines and Sex and Gender in Research

Cell line source(s)	EBY100 yeasts
Authentication	was not authenticated
Mycoplasma contamination	not tested for mycoplasma
Commonly misidentified lines (See ICLAC register)	None

Flow Cytometry

Plots

Confirm that:

☒ The axis labels state the marker and fluorochrome used (e.g. CD4-FITC).

☒ The axis scales are clearly visible. Include numbers along axes only for bottom left plot of group (a 'group' is an analysis of identical markers).

☒ All plots are contour plots with outliers or pseudocolor plots.

☒ A numerical value for number of cells or percentage (with statistics) is provided.

Methodology

Sample preparation	EBY100 yeasts were pelleted by centrifugation 30s at 13,300 RPM and resuspended into cold PBS-0.5%BSA for flow cytometry.
Instrument	BD FACSAria II
Software	BD FACSDiva V.8 (trademark of BD)
Cell population abundance	Enriched hits are propagated for the next round of sorting but difficult to know purity of hits at each stage
Gating strategy	Gate on majority of events, gate on singlets, relevant controls always used include unstained cells, cells with only each secondary reagent, and cells stained with everything yet not exposed to antigen. WT scFv was used as a control for gating to isolate weaker, moderate, and stronger binders.

☒ Tick this box to confirm that a figure exemplifying the gating strategy is provided in the Supplementary Information.
